# Mapping CD4+ T cell diversity in CSF to identify endophenotypes of multiple sclerosis

**DOI:** 10.1093/braincomms/fcaf231

**Published:** 2025-06-10

**Authors:** Tadhg Crowley, Jessy Chen, Kamil S Rosiewicz, Lea Jopp-Saile, Gesche Herold, Charlotte Biese, Cornelius Fischer, Janis Kerkering, Marlen Alisch, Friedemann Paul, Volker Siffrin

**Affiliations:** Experimental and Clinical Research Center (ECRC), Charité—Universitätsmedizin Berlin and Max Delbrück Center or Molecular Medicine in the Helmholtz Association, Berlin 13125, Germany; Experimental and Clinical Research Center (ECRC), Charité—Universitätsmedizin Berlin and Max Delbrück Center or Molecular Medicine in the Helmholtz Association, Berlin 13125, Germany; Department of Neurology and Experimental Neurology, Charité—Universitätsmedizin Berlin, Berlin 13353, Germany; Berlin Institute of Health (BIH), Charité Universitätsmedizin Berlin, Berlin 10178, Germany; Experimental and Clinical Research Center (ECRC), Charité—Universitätsmedizin Berlin and Max Delbrück Center or Molecular Medicine in the Helmholtz Association, Berlin 13125, Germany; Faculty of Biosciences, University of Heidelberg, 69120 Heidelberg, Germany; Heidelberg Institute for Stem Cell Technology and Experimental Medicine (HI-STEM gGmbH), 69120 Heidelberg, Germany; Division of Stem Cells and Cancer, German Cancer Research Centre (DKFZ), 69120 Heidelberg, Germany; Max Delbrück Center for Molecular Medicine in the Helmholtz Association, Berlin Institute for Medical Systems Biology, Berlin 10115, Germany; Experimental and Clinical Research Center (ECRC), Charité—Universitätsmedizin Berlin and Max Delbrück Center or Molecular Medicine in the Helmholtz Association, Berlin 13125, Germany; Experimental and Clinical Research Center (ECRC), Charité—Universitätsmedizin Berlin and Max Delbrück Center or Molecular Medicine in the Helmholtz Association, Berlin 13125, Germany; Berlin Institute of Health (BIH), Charité Universitätsmedizin Berlin, Berlin 10178, Germany; Experimental and Clinical Research Center (ECRC), Charité—Universitätsmedizin Berlin and Max Delbrück Center or Molecular Medicine in the Helmholtz Association, Berlin 13125, Germany; Experimental and Clinical Research Center (ECRC), Charité—Universitätsmedizin Berlin and Max Delbrück Center or Molecular Medicine in the Helmholtz Association, Berlin 13125, Germany; Experimental and Clinical Research Center (ECRC), Charité—Universitätsmedizin Berlin and Max Delbrück Center or Molecular Medicine in the Helmholtz Association, Berlin 13125, Germany; Experimental and Clinical Research Center (ECRC), Charité—Universitätsmedizin Berlin and Max Delbrück Center or Molecular Medicine in the Helmholtz Association, Berlin 13125, Germany; Department of Neurology and Experimental Neurology, Charité—Universitätsmedizin Berlin, Berlin 13353, Germany

**Keywords:** multiple sclerosis, experimental neuroinflammation, chronic neuroinflammation, T cell subsets, cerebrospinal fluid

## Abstract

Multiple sclerosis (MS) is a chronic inflammatory CNS disease with heterogeneous manifestation. Prognostic markers for early classification of MS are currently under investigation. Higher diagnostic resolution of cerebrospinal fluid (CSF) has the potential to contribute significantly to patient stratification, which should be especially important for a subgroup of patients with high risk to convert to a progressive disease course. This study aimed to determine whether spectral flow cytometry of CSF cells could identify pathogenic CD4+ T cell subset in MS. Using a two-step approach, we designed a marker panel informed by publicly available transcriptomic datasets from early human MS and our own single-cell RNA sequencing (scRNA-seq) in acute and chronic experimental autoimmune encephalomyelitis (EAE), a murine MS model. Notably, chronic (‘phase’) markers such as *Il7r* and *Ramp3* (associated with memory T cells), *Itgb1* (integrin beta-1) and anti-apoptotic genes like *Dnaja1*, *Hsph1* and *Jun/AP-1* were enriched in EAE. These markers reflect pro-survival signalling and tissue-residency characteristics, including *CXCR6*, *CD69* and *Bhlhe40*, which suggest an adaptation of CD4+ T cells towards persistent neuroinflammatory responses in chronic EAE. This phase-specific marker profile highlights CD4+ T cells as both indicators and contributors to disease progression in EAE. Translating these findings to MS datasets, we found an enrichment of phase-specific markers in CSF cells. Spectral flow cytometry in an independent MS cohort revealed distinct memory and effector T cell subsets, indicating unique CSF signatures in MS. This study underscores the heterogeneity and dynamic changes of CD4+ T cells detectable by spectral flow cytometry, enhancing diagnostic resolution of CSF cells and informing more precise therapeutic strategies for MS.

## Teaser

Crowley *et al*. explored CD4+ T cell heterogeneity in experimental autoimmune encephalomyelitis and multiple sclerosis (MS). Using single-cell RNA sequencing and spectral flow cytometry, they identified distinct phase- and cluster-specific markers. These findings enhance diagnostic resolution and highlight potential endophenotypes, paving the way for personalized therapeutic strategies in MS.

## Graphical Abstract

**Graphical Abstract:**
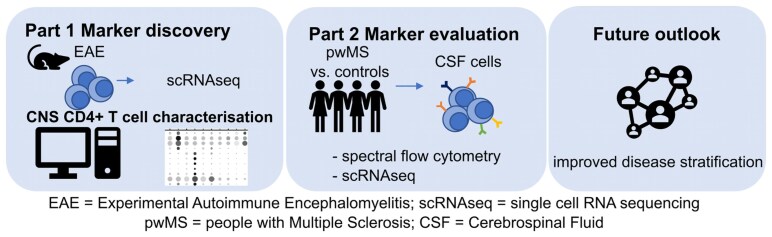

## Introduction

Multiple sclerosis (MS) is the most common chronic inflammatory disease of the central nervous system (CNS) leading to disability in young adults.^1,2^ The disease is thought to be initiated by peripheral invading immune cells. T cells can be identified in MS lesions early on and throughout the disease course,^3^ and are thought to be implicated in the complex pathophysiology of MS next to B cells.^4,5^ CD4+ T cells are the most abundant cell population in the cerebral spinal fluid (CSF) and were shown to be enriched in MS.^6^ Pathogenic T cells are considered one of the earliest to breach the blood-brain barrier in nascent demyelinating lesions.^7^ The animal model experimental autoimmune encephalomyelitis (EAE) serves to understand the transmigration processes of inflammatory T cells into the CNS parenchyma. It has been shown that CNS pathology was initiated by CNS-infiltrating myelin-specific CD4+ T cells^8^ and carried on to the development of clinical EAE by T cell activities dependent on the Th17 key transcription factor RORyt.^9^ Pathogenic T cells isolated from the CNS demonstrated significant changes compared to typical Th17 cells, notably losing IL-17A production and shifting to a Th1-like (ex-Th17) phenotype.^10^ This phenotype is associated with gene expression patterns enriched in genetic risk variants linked to MS.^11^ However, it remains unclear how CD4+ T cells acquire the capacity to home to the CNS and why focal inflammation persists over extended periods in MS—one correlate of progressive MS. Over the last year, numerous single-cell studies have underlined the diagnostic potential of in-depth T cell phenotyping not only in MS.^12,13^ These data demonstrate the accumulation of tissue-resident memory T cells (TRMs) with characteristic gene expression profiles,^13^ which were also suggestive as diagnostic marker to identify pre-clinical MS.^12^ An in-depth analysis using single-cell RNA sequencing of CSF T cells and integrative modelling identified T cell subsets with a shared TRM signature (*CXCR6, ZFP36L2, DUSP1* and *ID2*) tracking CNS-resident immune involvement in MS.^14^ Indirect evidence from studies on peripheral blood mononuclear cells in MS patients treated with Natalizumab (NTZ)—a drug believed to block the transmigration of pathogenic T cells into the CNS—suggests that this treatment may lead to an accumulation of these pathogenic T cells in the peripheral blood. Another study integrated several single-cell studies from CSF and CNS tissue from early MS disease stages, non-inflammatory controls and CSF from patients efficiently treated with NTZ. They found that CD4+ T cells with a distinct transcription factor profile (Runx3 + Eomes + T-bet−) and high levels of granzyme K and CCR5, particularly in Th17.1 cells, exhibit brain-homing abilities. Interestingly, this subset decreases in blood and accumulates in cerebrospinal fluid of untreated patients, with levels reversing after NTZ treatment.^15^

Our study aimed to set up a multicolour panel for spectral flow cytometry, a cost-effective method with the potential to be implemented into routine diagnostics with an emphasis on CD4+ T cell markers which have been associated with chronic disease progression. Next to available transcriptomic datasets of early human MS, we employed single-cell RNA sequencing (scRNA-seq) to analyse CD4+ T cell subsets in acute and chronic EAE. Findings were then translated to human MS transcriptomic datasets, confirming the enrichment of our identified cluster markers in CSF cells. Finally, we applied high-dimensional spectral flow cytometry to examine the surface expression of both established and novel CD4+ T cell markers in CSF from a discovery and extended cohort of early MS patients.

## Materials and methods

### Mice

All animals were raised and housed under SPF conditions in individually ventilated cages. Fluorescent reporter mice (B6.IL-17-EGFP_IFNg-YFP) were either derived from the creators^16^ or purchased from Jackson labs.^17^ All animal experiments were conducted according to the German Animal Protection Law. Experimental approval (G0009/16) was obtained from the State Office for Health and Social Affairs (‘LaGeSo’) in Berlin. Both male and female animals were used for EAE experiments.

### Experimental autoimmune encephalomyelitis (EAE)

To induce EAE in mice, 125 µg MOG_35–55_ (Charité Universitätsmedizin Berlin) emulsified in 400 µg *Mycobacterium tuberculosis* H-37Ra and complete Freund’s adjuvant (Sigma-Aldrich) was injected subcutaneously. This was followed by two intraperitoneal injections of 300 ng pertussis toxin reconstituted in Phosphate Buffered Saline (PBS, Gibco) at 2 and 24 h post-immunization. Mice were monitored daily for weight and clinical signs of EAE. These were scored as follows, 0.5, tail paresis; 1.0, tail plegia; 0.5, weak righting reflex; 1.0 hind limb paresis; 2.0 hind limb paralysis; 0.5 forelimb paresis and 1.0, forelimb paralysis. Mice reaching a total score of 3.5 or losing more than 20% of their initial weight were euthanized in line with animal welfare regulations. To induce remission of EAE, corticosteroid treatment (1.6 mg methylprednisolone in 200 µl 0.9% NaCl) was administered i.p. for 3–5 consecutive days when clinical signs exceeded a score of 2.^18,19^ All animal experiments had been approved by local authorities (G0009/16, Landesamt für Gesundheit und Soziales Berlin).

### Patient recruitment, ethics approval and consent to participate

The human subjects’ consent was obtained according to the Declaration of Helsinki. Ethical approval was obtained (internal number: EA1_023_15) by the ethical committee of Charité Berlin. PwMS (*n* = 21), including diagnoses of clinical or radiological isolated syndrome, and non-inflammatory control patients (*n* = 15) with idiopathic intracranial hypertension (IIH) or excluded inflammatory CNS disease were recruited during clinical routine diagnostics, where lumbar puncture was part of the diagnostic workflow. Ethical approval for this study was obtained (EA1_023_15) from the local Ethics Committee of the Charité. CSF samples from pwMS were included only if lumbar puncture was performed before steroid or other immunomodulatory treatments. Clinical details (age, sex) of pwMS and controls are listed in Supplementary Table S7.

### CNS T cell isolation

Mice were administered a lethal dose of a ketamine–xylazine mixture (ketamine 320 mg/kg; xylazine 5 mg/kg) intraperitoneally, followed by transcardial perfusion with 50 ml of ice-cold PBS. This procedure resulted in <1%, of contaminating T cells derived from the circulation, as verified using intravenously injected anti-CD45 fluorescently labelled antibodies in previous experiments. Brainstem and spinal cord tissues were removed, placed in ice-cold D-PBS (Ca2+, Mg2+, glucose, pyruvate, Gibco®) chopped into smaller pieces and transferred into C-tubes® (Miltenyi Biotec, Germany) containing enzyme mix 1 and 2 (for further details please refer to Miltenyi's Adult Brain Dissociation Kit Protocol). Tissue was homogenized using a gentleMACS® Octo Dissociator with Heaters, Miltenyi), programme 37C_ABDK_01. The homogenate was briefly centrifuged, resuspended in D-PBS then passed through a 100 µm cell strainer and centrifuged at 300×g and 4°C for 10 min. The resultant cell pellet was resuspended in 3.1 ml cold D-PBS mixed with 900 µl Percoll®, transferred to a 15 ml tube, overlaid with 4 ml cold D-PBS and centrifuged at 3000 × g for 10 min at 4°C without breaking. Following phase separation, the two top phases were aspirated and the cell pellet washed in cold D-PBS at 1000 × g for 10 min at 4°C. Erythrolysis was performed by resuspending the pellet in 10 ml of 1 × Red Blood Cell Removal Solution®, incubating for 10 min at 4°C and halting the reaction with 10 ml of cold D-PBS/BSA buffer (0.5% BSA, pH 7.2). A final wash at 300 × g for 10 min at 4°C yielded the single-cell suspension.

### *Ex vivo* T cell stimulation and immunostaining

For the cytokine production analysis, single-cell suspensions of CNS tissue were resuspended in RPMI-based cell culture medium (Gibco®), supplemented with 10% foetal calf serum, 1% penicillin/streptomycin, 1% GlutaMAX®, 1% HEPES. Cells were plated on anti-CD3 (BD Pharmingen, clone 145-2C11) and anti-CD28 (BD Pharmingen, clone 37.51)-coated culture plates and incubated overnight before immunostaining. Brefeldin A (BioLegend) was added 2 h after plating. Cells for surface analysis and single-cell sequencing were directly immunostained. Fc-blocking (BD Pharmingen) was performed for 15 min to reduce non-specific antibody bindings. Cells were then incubated for 10 min with the primary antibodies (CD11b, clone M1/70, BD Pharmingen; CD45, clone 30F11, Miltenyi and CD4, clone GK1.5, BioLegend) diluted in D-PBS/BSA buffer. For intracellular staining, cells were fixed in 2% PFA for 20 min washed in PBS, permeabilized in a saponin buffer (0.5% saponin, 0.5% BSA, pH 7.6), and stained with the antibodies against IFN-γ (clone XMG12, Biolegend), GM-CSF (Invitrogen) and IL-17A (clone eBio17B7, eBioscience). Stained cells were washed in PBS/BSA buffer before analysis.

### FACS-sorting

CNS cell suspensions were immunostained for cell surface antigens (see above) and sorted on a BD FACSAria™ II instrument. CD4^+^ T cells were gated by f size and granularity (FSC/SSC), single cells (FSC-H/FSC-W), PI^neg^, CD45^hi^, CD11b^neg^ and CD4^+^ cells. Sorted cells were collected directly into pre-cooled culture medium.

### Single-cell transcriptome analysis

To perform single-cell transcriptome analysis of CNS CD4+ T cells, we isolated CD45^hi^CD11b^−^CD4^+^ T cells by FACS-sorting from acute EAE (two samples, each two mice) and chronic EAE (two samples, each four mice). On average, using FACS, 180 000 cells from the acute phase and 70 000 cells from the chronic phase were isolated per replicate for scRNA-seq. Libraries were prepared according to the manufacturer's instructions using the 10 × Genomics platform for the Single Cell 3-prime Gene Expression v3 workflow. Sequencing was performed on the HiSeq 4000 platform and demultiplexed using bcl2fastq v2.20.0. Alignment, filtering, UMI counting, barcode-counting and gene-barcode matrix generation were done with Cell Ranger (version 3.0.2, 10 × Genomics).

### Flow cytometry of human CSF cells

CSF samples were processed within 1 h after lumbar puncture. Samples were centrifuged at 300× g for 10 min. The resulting cell pellet was stained with a fluorophore-conjugated antibody mixture for 10 min (Table 1). Staining was stopped with 1 ml FACS buffer. After washing, cells were fixed with 2% formaldehyde solution at room temperature. Following another wash step, cells were resuspended in 200 µl FACS buffer for analysis. Cytometric measurements were performed using a Spectral Flow Cytometer (Cytek Aurora®).

**Table 1:** Monoclonal antibodies utilized for flow cytometry analysis of human CSF cells

Biolegend	#Catalog	Clone	Dilution	Marker selection rationale (cluster/phase association or lineage)
CCR5	313711	HEK/1/85a	1:40	C0, C1 phase markers
CD29 Itgb1	303023	TS2/16	1:40	C3 cluster marker; C0, C2 phase marker
PD1	329949	EH12.2H7	1:40	C0 phase marker
CD8	300935	HIT8a	1:40	Lineage marker
CD45RO	304233	UCHL1	1:40	C1, C2 phase markers
CD4	317439	OKT4	1:40	Lineage marker
CD137	309843	4B4	1:40	
CD74	326807	LN2	1:40	C2 cluster/phase marker
IL7R	351313	A019D5	1:40	C0 phase marker
CD25	302607	BC96	1:40	C1 phase marker
CD69	310929	FN50	1:40	C1 phase marker
GITR	371205	108-17	1:40	C0, C2 phase marker
Streptavidin	405227		1:100	secondary antibody
CD103	350220	Ber-ACT8	1:200	C0, C1, C2 phase marker
KLRG1	138421	2F1/KLRG1	1:100	C2 cluster marker
CD19	302271	HiB19	1:40	Lineage marker
**BD**				
CD45	563791	563791	1:40	Lineage marker
CD16	612945	612945	1:40	Lineage marker
CD3	612896	612896	1:40	Lineage marker
CD14	612764	612764	1:40	Lineage marker
HLA-DR	612981	612981	1:40	Lineage marker

### CSF protein analysis

Results of CSF protein analysis were obtained from routine diagnostics at Labor Berlin—Charité Vivantes GmbH. IgG in the gel were separated electrophoretically based on their acidic and basic amino acid residues. Each IgG migrates in the electric field until the surrounding pH value corresponds to its isoelectric point and can be detected with a specific antiserum as a precipitation, which is visible for clonally expanded IgG as a band. Albumin, IgG, IgA and IgM were measured by nephelometry. Quotient schemes were calculated using the Reiber method.^20-22^

### EAE single-cell transcriptomics analysis and statistics

Count matrices were analysed in the R package, Seurat.^23,24^ Cell Ranger data were imported using the ‘Read10X’ function. Empty droplets and potential doublets were excluded by filtering for cells expressing more than 850 genes but fewer than 2800 genes. Contaminating macrophages were removed by excluding cells with detectable expression of P2ry12, Tmem119, Itgam, H2-Ab1, H2-Aa, Siglech, Ly6d, Cd209a, Cd209d or Lyz2. To exclude dead or dying cells, we filtered out those with mitochondrial gene expression exceeding 8% of total gene expression. Post-filtering, 15 253 cells (Acute 1: 3039, Acute 2: 4240, Chronic 1: 3445 and Chronic 2: 4529) passed quality control, with an average of 5000 reads/cell.

To integrate datasets across replicates and disease conditions, log-normalization was applied to each dataset individually, and the top 3000 variable features were identified. Seurat's IntegrateData function was used to combine datasets with default parameters. Clustering was performed based on the first 30 dimensions, using the Louvain algorithm. Following inspection of dimensionality by elbow plots of the first 50 dimensions, principal component analysis, clustering and dimensionality reduction via UMAP were performed on the first 20 dimensions. Cluster markers were identified using the FindConservedMarkers function to determine constant markers across disease phases. Clustering resolution was adjusted to the point where known cell-type-specific markers were identified in single clusters. Phase-specific markers were identified using the FindMarkers function to compare clusters between disease states. An adjusted p-value cut-off of p.adj < 0.05 was applied, and genes meeting this were analysed further using Ingenuity Pathway Analysis.

Publicly available single-cell sequencing data was downloaded from Zenodo (https://zenodo.org/records/6910635). Single-cell sequencing matrices were imported and Seurat Objects created using the CreateSeuratObject function (Seurat v5.0.1). Data were subsetted to CD4+ cells based on the provided metadata. To assess the enrichment of markers obtained from our mouse dataset, Seurat's AddModuleScore function was used to compute an enrichment score for non-mitochondrial and ribosomal mouse genes converted to their human homologues.

### CSF cells flow cytometry data analysis

Flow data was analysed using FlowJo® for manual gating or pre-processed for unbiased cluster analysis using Seurat. For manual gating, a minimum of 100 cells per CSF sample was required for downstream analysis. Compensated and FlowJo-normalized data were refined to include only lymphocytes. Data were loaded and processed using R (v4.3.0). Cells below a mean fluorescence intensity value of 1023 were filtered out to ensure data quality. Data were rescaled and centred before determining the principal components (PCs) across *n*–1 dimensions, where *n* was the maximum number of dimensions. Given that sample analysis spanned multiple days, batch correction was applied using Harmony (v0.1.1)^25^ to minimize variability in the PC space. Each sample was treated as an individual batch, and the integrated PC space was used for downstream analysis. A shared nearest neighbour graph (*k* = 20) was constructed as implemented in Seurat (v5). Clusters were identified using modularity-based clustering (Louvain algorithm^26,27^) and visualized with uniform manifold approximation and projection (UMAP).^28^ For a minimum distance of 0.3 and 30 neighbouring cells were used to enhance visualization of local structures in the dataset. To specifically focus on CD4+ T cells, we refined our data to include markers relevant to T cell lineage (KLRG1, CD29, CD25, CD103, CD154, IL7R, PD1, CD137, Il1R1, HLA-DR, CD69, CCR5, CD45RO and GITR). Clustering and dimensionality reduction were rerun on this refined dataset. Plots were generated using ggplot2 v3.4.2.^29^ Statistical analysis of manually gated flow data was performed using Graphpad Prism (Version 9.0, GraphPad Software, San Diego, CA, USA). Values are expressed as mean ± SEM. Statistical tests included Kruskal–Wallis, Dunn's multiple comparison, Mann–Whitney U, *t*-test, or one-way ANOVA, as appropriate (indicated in figure legends; *P* < 0.05 was considered statistically significant).

## Results

### ScRNA-seq of CNS CD4+ T cells in acute and chronic EAE identifies five subsets

**Figure 1:**
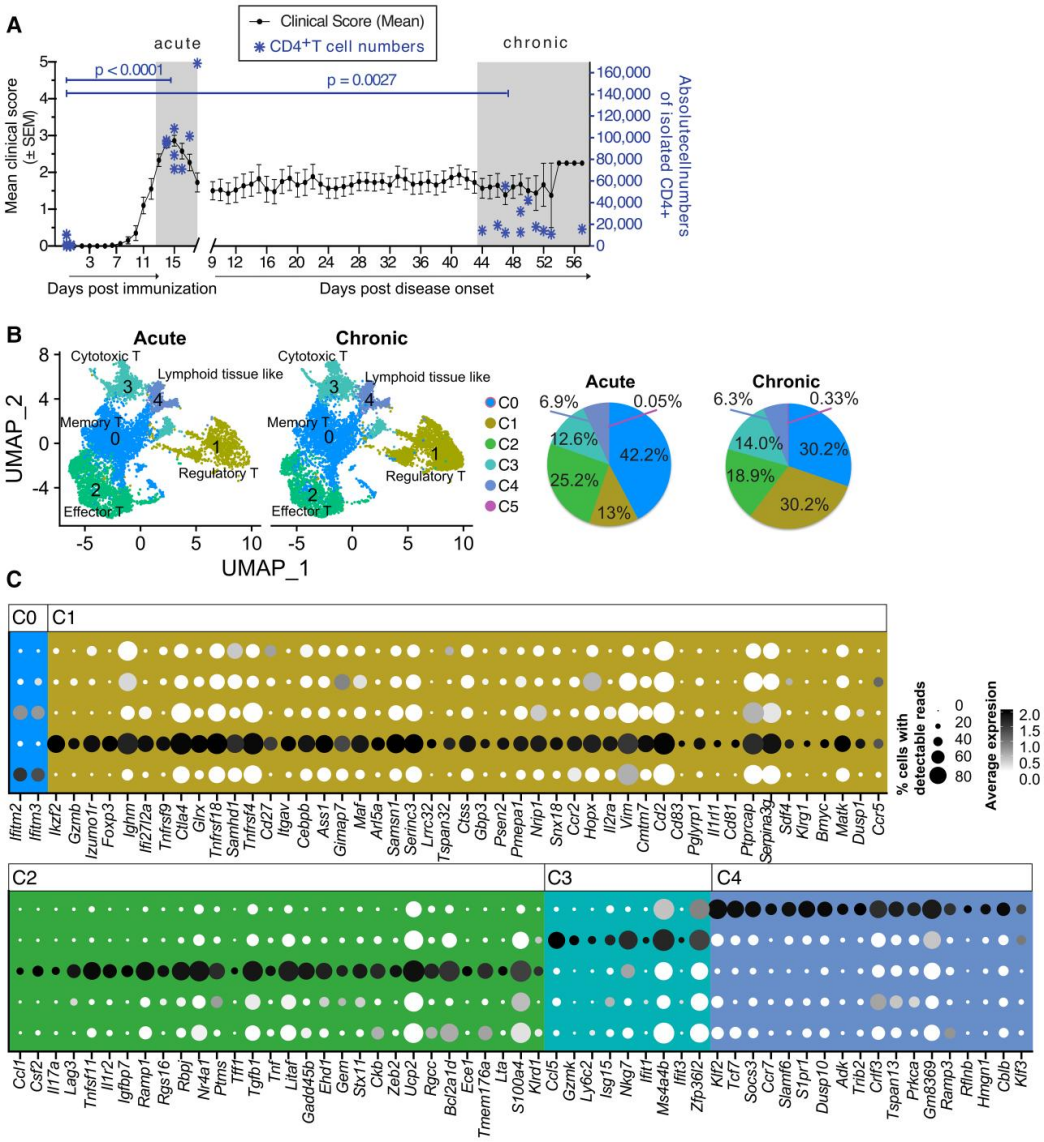
**scRNA-seq of CNS CD4+ T cells in acute and chronic EAE reveals five subsets.** (**A**) Time course of the mean clinical EAE score (±SEM, left *y*-axis) based on data from at least three independent experiments, with individual mouse scores synchronized to the first incidence of a clinical score ≥1. Asterisks denote absolute CD4+ T cell counts recorded by FACS (right y-axis; CD45hi CD11b− CD4+ T cells) in the CNS, which were significantly higher during both the acute and chronic EAE phases compared to the naïve control group. Sample sizes: control (*n* = 21), acute EAE (*n* = 10) and chronic EAE (*n* = 11). Statistical analysis was conducted using the Kruskal–Wallis test and Dunn's multiple comparison test. (**B**) UMAP visualization of Seurat-derived cell clusters using nonlinear dimensionality reduction, identifying six clusters (0–5, color-coded) in acute and chronic EAE (pooled data from two independent samples; the number of mice used in the scRNA-seq experiment is *n* = 4 (2 + 2) for acute EAE and *n* = 8 (4 + 4) for chronic EAE, resulting in on average 180 000 cells from the acute phase and 70 000 analysed cells from the chronic phase sorted and sequenced per replicate). (**C**) Dot plot showing output from IPA's Biomarker tool applied to Seurat's FindMarkers results (threshold: Fold Change > 1) to identify biologically relevant genes marking each cluster (positive expression). Clusters C0–C4 are color-coded as follows: C0: red, C1: yellow, C2: green, C3: blue, C4: purple. The fully resolved gene abbreviations are provided in Supplementary Table S8.

In EAE, myelin-specific CD4+ T cells elicit an autoimmune reaction within the CNS leading to ascending paralysis that begins 10–14 days after subcutaneous immunization with myelin oligodendrocyte glycoprotein in conjunction with adjuvants. During the acute phase of EAE, clinical signs peak 3–4 days after onset. For single-cell transcriptome analysis, mice selected for analysis at the peak of the disease had a clinical score ranging from 3.0 to 3.5 (Fig. 1A, left axis). Chronic EAE was defined as 30 or more days after the onset of clinical signs, with chronic EAE animals showing a score range of 0.75–2.75. The CNS-isolated CD4+ T cell counts were significantly elevated at the peak of the disease (median: 95 565 cells/mouse) compared to non-immunized control animals (median: 364 cells/mouse). Although CD4+ T cell numbers declined in chronic EAE (median: 15 659 cells/mouse; Fig. 1A, right axis, statistical analysis in Supplementary Fig. S1B), they remained consistently higher than in control animals. FACS analysis (Supplementary Fig. S1A) revealed that a substantial proportion of CNS effector T cells expressed the cytokines IFN-γ, GM-CSF and IL-17A in both acute and chronic EAE. Next, we FACS-sorted CD45^hi^CD11b^−^CD4^+^ T cells and used the droplet-based 10 × Genomics Chromium approach to examine disease states. ScRNA-seq was performed on the HiSeq 4000 platform. Using unbiased clustering based on the top variable genes present in both conditions, we identified six clusters overall (referred to as Cluster 0–5 or C0–C5) with C0 being the largest and C5 the smallest (Fig. 1B). No clusters were found exclusively in chronic EAE. Still, cluster-specific shifts in proportion were observed for clusters C0–C3. C1, identified as regulatory T cells, increased in proportion in chronic EAE (from 13% in acute EAE to 30.2% in chronic EAE). Conversely, the chronic EAE Cluster C0 showed a size reduction (from 25.2% in acute EAE to 18.9% in chronic EAE; Fig. 1B). Clusters 3, 4 and 5 were comparable in proportion between the two different disease states.

Differential lineage marker analysis confirmed the accuracy of our positive sorting on CD4+ T cells (Supplementary Fig. S1C). Due to very low cell numbers in C5 (acute: 4 cells, or 0.05% in acute EAE), a reliable in-depth analysis of this subset was not possible. Consequently, all further analysis focused on C0–C4. We used Seurat's FindMarkers function and Ingenuity® Pathways Analysis (IPA) Biomarker analysis tool, which enabled the cluster differentiation into memory (C0), regulatory (Treg) (C1), effector (C2), cytotoxic (C3) and lymphoid-tissue-like (C4) phenotypes (Fig. 1C and Supplementary Fig. S1D). A complete marker list is available in Supplementary Table S1. In Clusters C0–C3, we observed an enrichment of tissue residency markers (*Cxcr6*, *Cd69* and *Bhlhe40*) in C0–C3 (Supplementary Fig. S1D). The C0 cluster was memory-like and lacked differentially expressed genes, with only an increased expression of innate immune receptors *Ifitm2* and *Ifitm3* could be found. The C1 Treg cluster was defined by a large set of differentially upregulated genes involved in Treg activation and effector functions that included cytotoxic potential. The conventional T effector subpopulation, C2, expressed genes involved in pro-inflammatory cytokine production, inhibition of proliferation, modulating cytokine/TCR signalling and a diverse set of co-stimulatory and exhaustion markers. C3 displayed the characteristic gene signature of Tbet-dependent cytotoxic CD4+ T cells, with machinery for antiviral responses and cytotoxicity. Cluster C4 exhibited a lymphoid-tissue–typical gene expression profile—closely resembling that of central memory T cells. Additional information on previously reported genes in the context of T cells and neuroinflammation is available in Supplementary Table S2.

### Cluster-specific adaptations dominate between acute and chronic EAE, and pro-survival prevails across subsets

**Figure 2:**
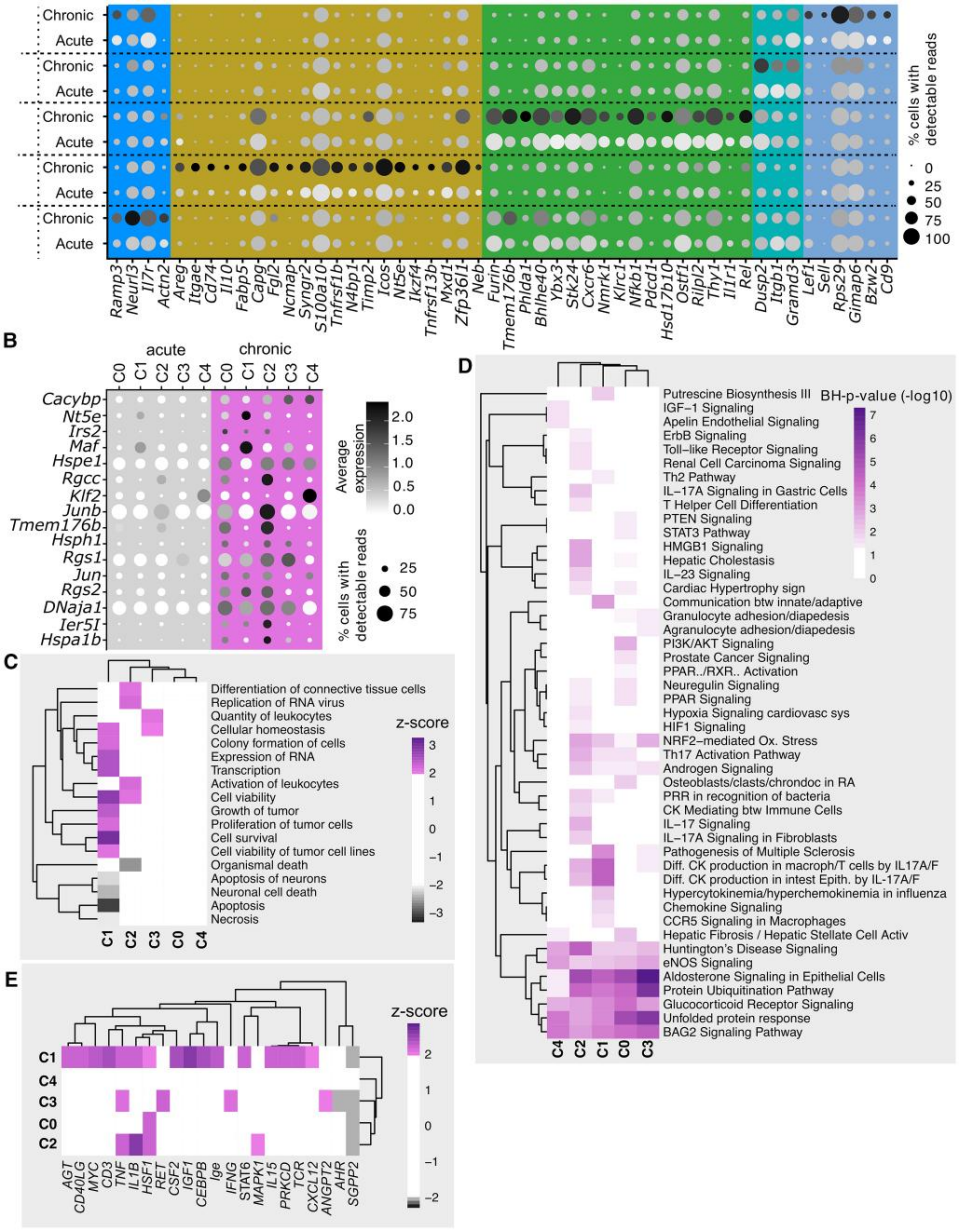
**Cluster-specific adaptations between acute and chronic EAE highlight pro-survival signals across subsets.** (**A**) Cluster-specific gene enrichment changes in the chronic phase of EAE. Spot size represents the proportion of cells in each cluster expressing the gene, while greyscale intensity indicates the average expression level within the cluster. Clusters are color-coded as follows: C0: red, C1: yellow, C2: green, C3: blue, C4: purple (legend provided for reference). The ‘acute’ dataset corresponds to the acute EAE phase, while the ‘chronic’ dataset represents the chronic EAE phase, as defined in Fig. 1 (pooled data from two independent samples; the number of mice used in the scRNA-seq experiment is *n* = 4 (2 + 2) for acute EAE and *n* = 8 (4 + 4) for chronic EAE, resulting in on average 180 000 cells from the acute phase and 70 000 analysed cells from the chronic phase sorted and sequenced per replicate). The fully resolved gene abbreviations are provided in Supplementary Table S8. (**B**) Genes enriched across multiple clusters (pan-clusters) during the chronic phase of EAE (based on scRNA-seq data set as described in A). The fully resolved gene abbreviations are provided in Supplementary Table S8. (**C**) Ingenuity Pathway Analysis (IPA) of differentially expressed genes between disease phases predicts activation of pathways related to cell survival and apoptosis [magenta: chronic phase, grey: acute phase; (based on scRNA-seq data set as described in A)]. Z-scores reflect the expression direction versus known effects; no statistical testing has been applied. (**D**) IPA of molecular functions and diseases affected between chronic and acute EAE based on differentially expressed genes, determined by cluster-wise comparisons of disease phases. Adjusted *P*-value threshold = 0.05 (based on scRNA-seq data set as described in A). IPA pathway enrichment analysis uses right-tailed Fisher's Exact Test and Benjamini-Hochberg (BH) correction. (**E**) IPA-predicted upstream regulators of differentially expressed genes between disease phases [magenta: chronic phase, grey: acute phase; (based on scRNA-seq data set as described in Fig. 2A)].

The persistence of T cells within the CNS is one of the main features of multiple sclerosis, but the mechanisms behind T cell persistence remain unclear. To identify factors involved in these processes, we analysed cluster-specific markers, or ‘phase markers’, which define chronic versus acute EAE for each of the identified CD4+ T cell subpopulations (Fig. 2A). In chronic disease, C0 showed increased expression of memory T cell markers such as *Il7r* and *Ramp3* in chronic disease. C1 upregulated genes associated with effector or cytotoxic Treg in chronic EAE. C2 showed an increase in tissue-resident marker expression and demonstrated metabolic changes in chronic EAE. C3 showed selective upregulation of integrin beta-1 (*Itgb1*) and other genes upregulated in chronic EAE. For C4, we observed an upregulation of typical lymphoid-tissue-related genes. Additional information on previously reported genes in the context of T cells and neuroinflammation is available in Supplementary Table S3.

A comparison of disease phases across the entire CD4+ T cell population revealed commonalities in metabolism and apoptotic signalling (Fig. 2B). When analysing genes enriched in the chronic phase of EAE across multiple CD4+ T cell subtypes, a smaller subset of genes emerged from the analysis. In particular, genes involved in anti-apoptotic signalling—e.g. *Dnaja1* (DnaJ homologue subfamily A member 1) and heat shock protein-coding genes, e.g. *Hsph1/Hspe1/Hspa1b* were upregulated in chronic EAE and have been previously reported as essential for the manifestation of clinical signs in EAE.^30^ Pathway analysis of cluster-specific differential gene expression demonstrated phase and cluster-specific pathway activation patterns (Fig. 2C). Notably, C1 (Treg) showed chronic phase enrichment in programmes associated with effector Treg function (Fig. 2C). For C1, IPA-predicted a relative decrease in pathways involved in neuronal apoptosis and cell death. C2 (Teff) displayed an increase in leukocyte activation signals and inflammatory signalling and diverse Th17-related pathways. IPA-based prediction of the molecular functions and regulated pathways from our cluster-specific pairwise comparisons showed an increase in survival pathways across all subsets in the chronic phase of EAE (Fig. 2D). We identified a unifying signature of anti-apoptotic signalling involving protein ubiquitination pathways, glucocorticoid receptor signalling pathways, heat shock responses and unfolded protein responses. Examining potential upstream inducers of these programmes, we identified HSF1 as a candidate regulator of anti-apoptotic and heat shock responses (Fig. 2E). The relative absence of SGPP2 (sphingosine 1 phosphate phosphatase 2) activity appears to be a unifying potential regulatory factor for all tissue resident clusters (C0–C3). Inflammatory cytokines are known to influence chronic EAE; here, we saw the influence of TNF in C1 to C3 and of IL-1b in C1/C2. In line with these observations, TCR stimulation and CD40L activation are potential inducers of the observed chronic EAE changes in C1.

These results indicated that CD4+ T cell subtype changes in persistent neuroinflammation include adaptation towards a tissue-resident phenotype in C0–C3 and a specialized, antagonistic effector function in C1 and C2. C4 exhibits a more pronounced central memory phenotype. A common pattern of increased anti-apoptotic and pro-survival programmes can be seen in all clusters. Collectively, these findings indicate a role for these markers in the CNS persistence of CD4+ cells beyond the acute inflammatory period.

### Compartment-specific and disease-associated enrichment of EAE CD4+ T cell cluster and phase markers in MS

**Figure 3:**
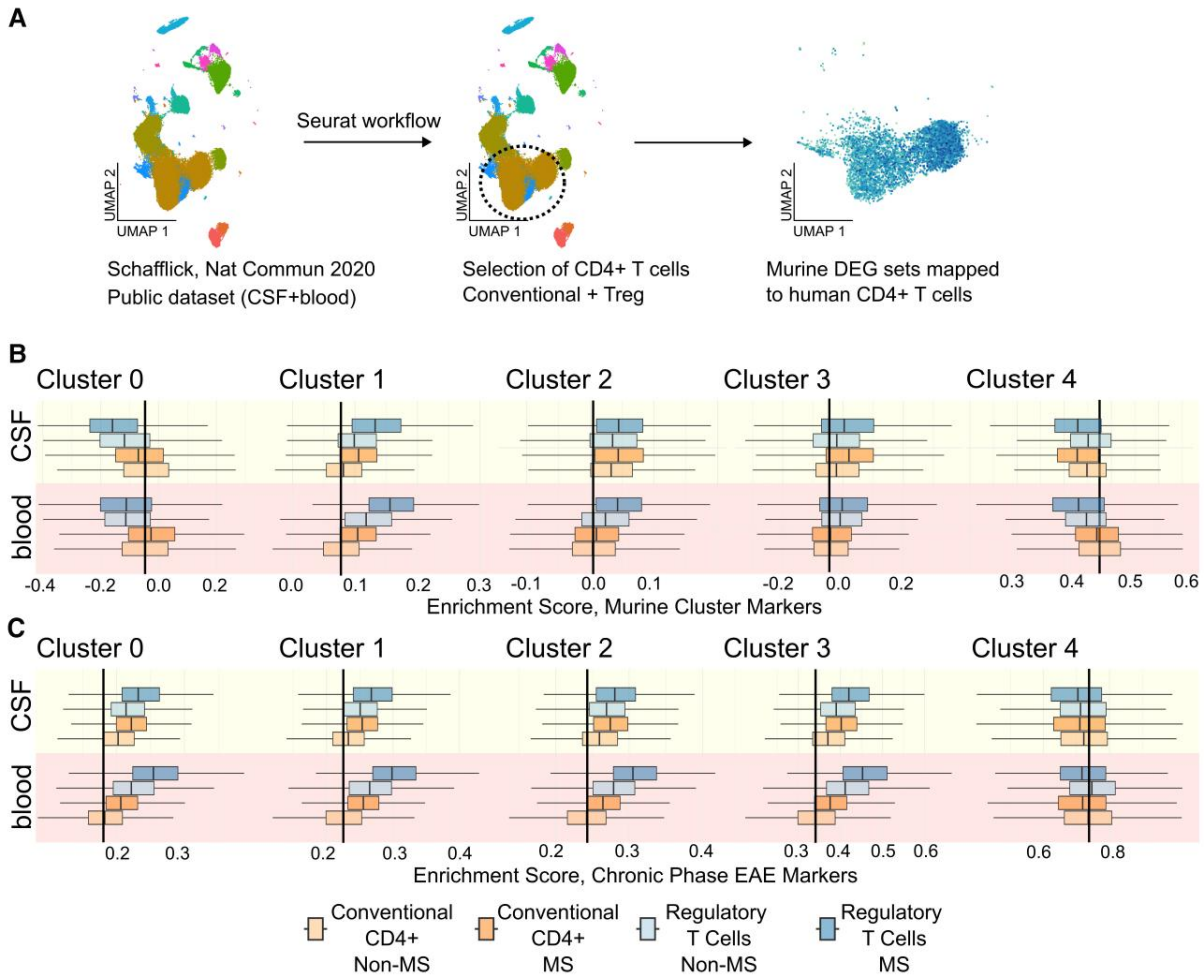
**Mapping EAE-identified cluster and phase markers onto human MS scRNA-seq data.** (**A**) Schematic overview of the workflow used to integrate murine and human scRNA-seq datasets. A publicly available single-cell dataset (reproduced from Schafflick *et al*.^1^) from human cerebrospinal fluid (CSF) and blood of early pwMS (pwMS vs control; blood (*n*): 5 versus 5; CSF (*n*): 4 vs 4) was analysed to assess the expression of EAE-identified, cluster-specific markers. After selecting CD4+ T cells, murine cluster marker sets (C1–C4, as outlined in Supplementary Table S4) were mapped onto human MS and control samples to identify enriched genes. (**B**) Box and whisker plots showing the distribution of enrichment scores of cluster markers from clusters C0 to C4 in human CD4+ T cells, subdivided into conventional helper T cells (Tcon) and regulatory T cells (Treg). C0 markers were enriched in both CSF and blood CD4+ conventional T cells, while C1 markers showed a notable enrichment in blood-derived Tregs, consistent with their characterization as markers of memory and regulatory cells (see also Supplementary Fig. S2A). MS = multiple sclerosis, ctrl = control. Statistical analyses were performed using the Mann–Whitney U test, with Bonferroni correction applied for multiple comparisons (see Supplementary Table S5 for full details). (**C**) Box and whisker plots showing the enrichment of chronic phase DEGs from clusters C0 to C4 in the human dataset. Phase-defining markers for Clusters C0–C3 were enriched in both CSF and blood CD4+ T cells (Tcon and Treg) in pwMS compared to controls. In contrast, C4 markers were similarly distributed between the two groups, suggesting a lack of disease-specific association (see also Supplementary Fig. S2B). Statistical analyses were performed using the Mann–Whitney U test, with Bonferroni correction applied for multiple comparisons (see Supplementary Table S6 for full details).

In the next step, we analysed a publicly available single-cell RNA-seq dataset integrating CSF and blood samples from early pwMS to assess the expression of cluster-specific and differentially upregulated genes identified in EAE (Fig. 3A). Using the Seurat workflow, we selected conventional CD4+ T cells (Tcon) and regulatory T cells (Treg) from the Schafflick *et al*. dataset and mapped the murine differentially expressed genes (DEGs) sets onto the human data (Supplementary Fig. S2). The calculated enrichment scores for both the cluster markers (Fig. 3B) and phase markers (Fig. 3C) provide a quantitative measure of whether a predefined set of DEGs from one dataset is statistically over-represented in another, allowing us to assess the relevance of EAE-identified signatures in human CD4+ T cell samples.

The cluster markers (Supplementary Table S4) were mapped separately onto conventional (Tcon) and regulatory (Treg) CD4+ T cells in the human dataset (Supplementary Fig. S2A), further differentiating between MS and control samples. For the cluster markers (Fig. 3B; Supplementary Fig. S2B), C1 (Treg) markers showed an enrichment in Tregs (using the Tcon as reference) and further exhibited disease-specific enrichment in both the CSF and blood of pwMS compared to controls. C0 markers were reduced in Tregs across both compartments (blood and CSF, referenced to Tcon) but did not show strong disease-related differences. C2 (effector) markers increased expression in both Tcon and Treg populations in the CSF compared to blood Tcon, with an additional enrichment in blood-derived MS Tregs. C3 markers (cytotoxic) were similarly distributed in Tcon and Treg populations in the CSF but were reduced in Tcon cells in the blood. C4 cluster markers (lymphoid-tissue-like) showed no significant differences related to disease status or compartment specificity. Overall, Tregs in MS, particularly in CSF, showed strong enrichment for the identified cluster markers, suggesting a disease-specific regulatory T cell phenotype.

We then performed a similar analysis for the phase markers (Fig. 3C, Supplementary Fig. S2C) that distinguished chronic from acute EAE in the human dataset (Supplementary Table S4). C4 phase markers showed no significant differences between Tcon and Treg populations in either the CSF or blood. In contrast, phase markers from C0 to C3 were enriched in Tcon and Treg MS samples compared to Tcon (blood) across both compartments, with the most pronounced enrichment observed in Tregs across all clusters in MS versus control samples.

Notably, Tregs showed greater enrichment than Tcon in the CNS compared to blood, along with clear disease-associated differences within both subsets. These findings suggest that EAE-derived phase markers (C0–C3) are relevant for the persistence of certain CD4⁺ T cell subpopulations in the CNS, reinforcing their involvement in chronic neuroinflammation. Consistent and significant enrichment of chronic phase markers in MS-derived Tregs and CD4⁺ cells—particularly in the CSF—further supports their role in disease progression.

### CD4+ T cell subset analysis in early MS distinguishes between a more acute versus chronic CSF signature

Building on our findings from the EAE model and publicly available MS datasets, we applied spectral flow cytometry to further characterize CD4+ T cell subsets in human CSF. Our marker panel was designed based on (i) cluster and phase markers identified in EAE (Table 1, Supplementary Table S4, Supplementary Fig. S1) and (ii) additional markers to distinguish non-CD4+ T cell populations, ensuring a comprehensive analysis. Using this approach, we investigated CNS T cell subsets in an independent cohort of pwMS and controls to assess differences in immune signatures between acute and chronic disease states.We recruited pwMS who were referred to our inpatient service due to clinical and MRI suspicion of chronic inflammatory disease, along with control patients diagnosed with IIH, a non-inflammatory condition, during routine diagnostics. We analysed CSF samples from a discovery cohort of 25 patients (*n* = 15 MS, *n* = 0 IIH).

**Figure 4:**
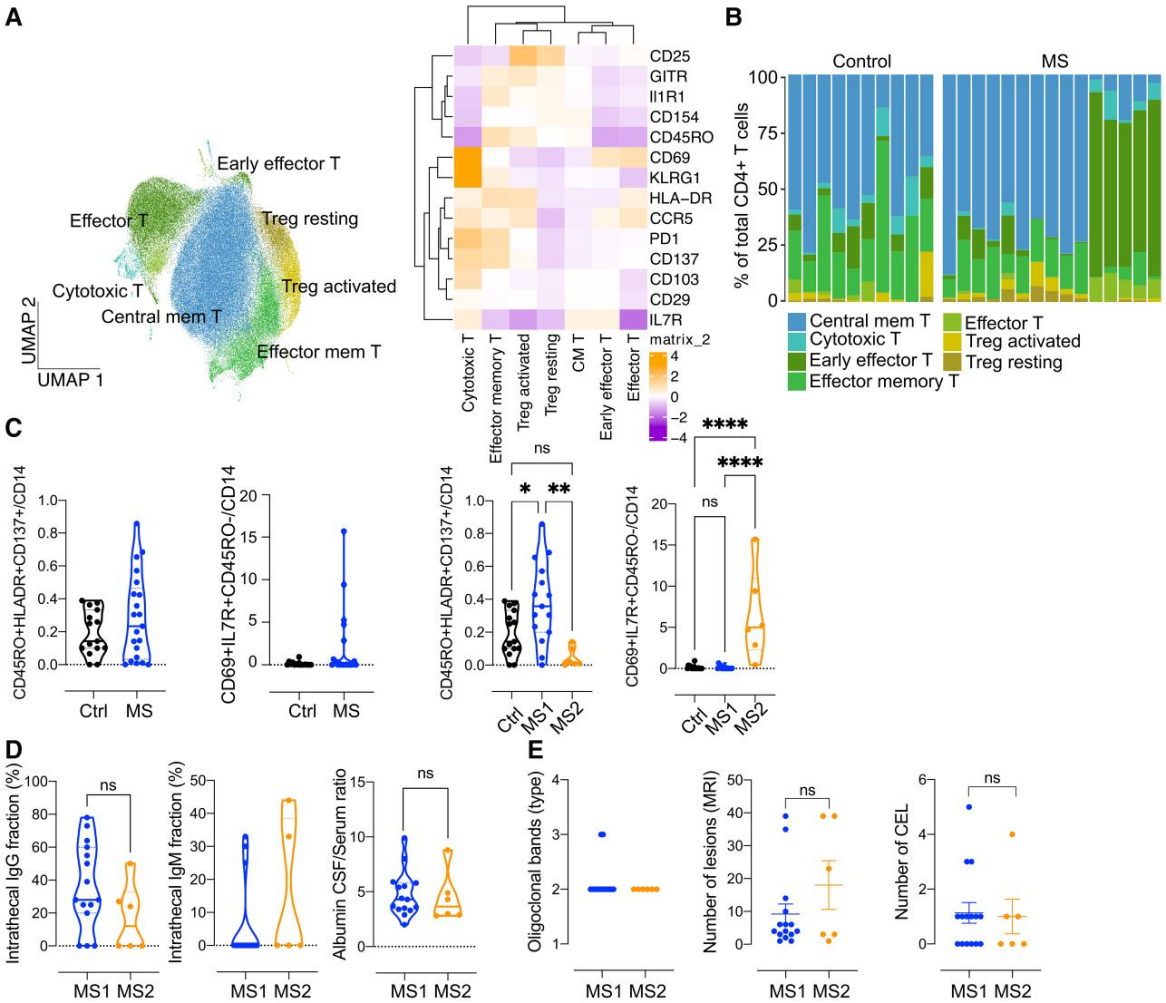
**CD4+ T cell subset analysis in early MS reveals distinct acute and chronic CSF endophenotypes.** As suggested, we have clarified the specific statistical test applied to each dataset in the figure. (**A**) UMAP visualization of CD4+ T cell clusters identified through Louvain clustering in Seurat. Heatmap displays the markers used for cluster annotation, with cluster labels including CD4+ T cells (T), central memory T cells (CM T) and regulatory T cells (Treg). Sample size MS = 15, control = 10. (**B**) Bar plot showing the distribution of CD4+ T cell subsets in individual patient samples, highlighting differences between MS and control groups. (**C**) Ratio of CD4+ T cell subset counts normalized to CD14+ monocyte counts, with values expressed as mean ± SEM. Graphs compare control and MS groups, as well as MS1 and MS2 subgroups, across various parameters (*y*-axis), highlighting differences in CD4+ T cell subset composition relative to stable CD14+ monocyte counts. Each data point represents one patient sample in groups as indicated. Statistical analysis was conducted using the Shapiro–Wilk test for normal distribution, followed by either *t*-test (MS versus ctrl) or one-way ANOVA (Ctrl versus MS1 versus MS2). Sample size MS = 21 (MS1 = 15, MS2 = 6), Control = 15. Significance levels are denoted as follows: ns, non-significant, *P < 0.05, **P < 0.01, ***P < 0.001 and ****P < 0.0001. (**D** and **E**) Clinical routine parameters, with values expressed as mean ± SEM. Types of oligoclonal bands are defined as follows: type 1 = none, type 2 = exclusive to CSF and type 3 = identical in both CSF and serum, with additional bands in CSF. CEL , contrast-enhancing lesion. Statistical analysis was conducted using the Shapiro–Wilk test for normal distribution, followed by either *t*-test (IgG; Alb ratio) or Mann–Whitney test (IgM; lesion number, CEL). Each data point represents one patient sample in groups as indicated. Sample size MS = 21 (MS1 = 15, MS2 = 6). Significance levels are denoted as follows: ns, non-significant, *P < 0.05, **P < 0.01, ***P < 0.001 and ****P < 0.0001.

Initial analyses were conducted in an unbiased manner with the Seurat workflow. Foetal calf serum files were batch-integrated with Harmony, ^25^ and CD45 + CD3 + CD4+ clusters were selected for a refined clustering based on activation markers. UMAP visualization showed distinct clustering of CD4+ T cell subpopulations (Fig. 4A) in both MS and non-inflammatory control patients. The identified subpopulations included resting Tregs (CD25^hi^IL7R^low^), activated Tregs (CD25hiCCR5 + GITR + HLADR+) and conventional T cells, including central memory T cells (CD45RO^low^IL7R^hi^), effector memory T cells (CD45RO^hi^PD1 + HLADR + CD137+), effector T cells (CD45RO-CCR5 + PD1 + IL7R^low^), early effector T cells (CD69 + CD45RO-CCR5+) and a cytotoxic/innate-like (KLRG1+) cluster of T cells (Fig. 4A). Individual patient analysis revealed differences in CD4+ T cell composition, showing either dominant memory/effector memory T cells or effector/early effector T cells in pwMS, which were not observed in the control cohort (Fig. 4B). Due to variability in CSF cell counts and the potential impact of low cell numbers on clustering algorithms, we verified these observations by manual gating in FlowJo® for both the discovery cohort and an extended cohort (MS = 6, controls = 5, totalling MS = 21 and controls = 15) (Supplementary Fig. S3A). We found that these differences were primarily driven by CD45RO and CD69 expression (Supplementary Fig. S3B). Despite considering batch effects, the observations remained consistent within the MS cohort, across different investigators, and over varying measurement days. There were no significant age variations in our cohort; females were more frequent in the control group (persons with IIH; Supplementary Fig. S3D).

Based on these findings, we categorized MS patients into two CSF signature groups: MS1, with a CSF signature favouring memory over effector T cells (Memory > Effector), and MS2, with a signature favouring effector over memory T cells (Effector > Memory). To determine whether this difference arose from a shift within CSF CD4+ T cell subsets or from additional invading CD4+ T cells, we calculated absolute counts of each T cell subpopulation and normalized these to CD14+ monocyte counts (Supplementary Fig. S3B) according to our previous work.^6^ We then analysed ratios of CD4+ T cell subsets to CD14+ counts in controls versus MS and MS1 versus MS2 patients, confirming the subset distribution observed with our algorithm (Fig. 4C).

We noted a trend towards increased Treg/CD14+ ratios within the MS1 subtype, while the MS2 subtype exhibited a higher ratio of CCR5+ Tregs (Supplementary Fig. S3C). Comparing CSF CD4+ T cell subsets to paraclinical CSF data, we found that the MS1 subtype had a trend towards higher intrathecal IgG production compared to the MS2 subtype (Fig. 4D). The albumin CSF/serum ratio, an indicator of blood-brain barrier integrity, showed no significant difference. Additionally, we observed no correlation between CSF/serum oligoclonal band IgG patterns and T cell signatures (Fig. 4D). PwMS showed no differences in disability, age, or sex at the time of CSF analysis (Supplementary Table S7), and MRI T2 lesion load, and contrast-enhancing lesions on T1 imaging did not differ between MS1 and MS2 (Fig. 4E).

## Discussion

Our comprehensive characterization of CD4+ T cells in experimental neuroinflammation and MS reveals that phase-specific markers distinguishing chronic states of neuroinflammation are detectable in the CSF of individuals with early MS. By integrating single-cell RNA sequencing and high-dimensional flow cytometry, we demonstrate that in-depth profiling of CD4+ T cell subsets can be effectively achieved, providing valuable insights beyond conventional diagnostic parameters. High-dimensional spectral flow cytometry, implemented as a feasible approach for routine diagnostics, identified distinct CD4+ T cell signatures in the CSF, potentially enhancing diagnostic resolution and advancing our understanding of disease heterogeneity. These findings underscore the potential of CSF cellularity analysis to capture unique cellular adaptations associated with neuroinflammation stages, which could facilitate the development of personalized endophenotype-driven therapeutic strategies in MS. While these results are exploratory, they pave the way for further studies with larger cohorts to strengthen correlations with prospective clinical data and refine endophenotype characterization.

Over recent years, several CSF scRNA-seq studies from pwMS have provided critical insights into the cellular landscape of the CSF. Notably, Ostkamp and colleagues^14^ investigated immune cells in CSF and CNS parenchyma of pwMS, comparing early disease stages, relapse and stable disease states under α4-integrin blockade via Natalizumab (NTZ). Their work revealed a tissue-resident phenotype across multiple lineages including T cells with shared expression of *CXCR6* and differential regulation of *CD103*, indicating cellular adaptations involved in tissue retention. In CD4+ T cells, CCR5^hi^ Th17.1 and CD4+ CD45RA+ T effector memory cells showed high tissue retention capacity. Our results build on this foundation, identifying phase markers for CD4+ T cell subsets with potential roles in chronic inflammation and neuroinflammatory processes in MS.

Several studies emphasize the role of specific CD4+ T cell subsets, particularly Th17 cell subsets, in MS. A recent study demonstrated that brain-homing Th17.1 cells were resistant to glucocorticoids and were elevated in the peripheral blood of NTZ-treated MS patients, while also predominating the CSF of early MS.^31^ In our study, by mapping EAE-derived markers to the work of Schafflick *et al*.,^32^ we identified cluster- and phase-specific alterations that suggest the utility of phase markers from C1 to C3 in pinpointing potential pathogenic T cells in MS. These findings align with our observation that specific markers enriched in EAE reflect similar immune adaptations in human MS, further supporting the use of EAE as a model to explore CD4+ T cell-related mechanisms in the pathophysiology of MS.

The advantage of single-cell sequencing lies in its ability to analyse the heterogeneity of immune cells with pathogenic potential. However, limitations such as cost and the need for large cell numbers constrain its applicability in routine diagnostics, especially when dealing with CSF, where rare cell numbers and interindividual variability make analysis challenging. Spectral flow cytometry, a recently upgraded flow cytometry technique, allows for the simultaneous analysis of up to 40 markers, enabling clustering at a resolution comparable to sequencing studies.^33,34^ Prior work has demonstrated that CSF immune cell composition holds prognostic value and could aid in treatment decisions.^6,35,36^ This technique has proven effective in recent studies investigating blood-derived mononuclear cells, including those examining pwMS treated with NTZ^37^ or dimethyl fumarate,^38^ to highlight potentially pathogenic immune cell subsets in a treatment-dependent manner. Our flow cytometry cohort revealed distinct CD4+ T cell signatures, showing the heterogeneity of inflammatory states in pwMS. The most notable differences were found in CD69+/CD45RO− (effector) and CD69−/CD45RO+ (memory) T cells, highlighting an underappreciated phasic aspect of inflammation in MS. While our study primarily focused on early active MS, the potential impact of relapse versus remission on CD4+ T cell subsets remains to be fully elucidated. CSF immune cell composition may change dynamically during different disease phases, with a potential shift in T cell activation states, tissue retention characteristics, and pro-inflammatory versus regulatory balance. Although our findings indicate distinct CD4+ T cell signatures in MS, it is currently unclear whether these signatures are phase-dependent or reflect a broader spectrum of immune responses. Notably, the MS1 subset in our study exhibited a CD4+ T cell composition more comparable to non-inflammatory controls, raising the possibility that our observed variations may correspond to distinct disease states or different immune adaptations within the same phase. Future studies with larger, longitudinally sampled cohorts will be critical to disentangle these effects and determine whether CSF CD4+ T cell profiles could serve as reliable markers for disease stage, relapse risk, or progression to more advanced MS phenotypes. Additionally, unbiased bioinformatics approaches, such as clustering algorithms and machine learning-based stratification, could help refine subgroup classification and validate the robustness of MS1 and MS2 groupings. However, these methods require significantly larger datasets to ensure statistical power and generalizability, particularly when distinguishing between disease phases and immune response variability. This will be interesting for rarer subsets, which may be underappreciated in our study, such as activated vs resting Tregs or cytotoxic CD4+ T cells.

MS1 and MS2 signatures did not correlate with baseline clinical presentations typically observed during the initial MS episode. However, conventional CSF antibody analysis revealed differences in oligoclonal bands, suggesting that CSF CD4+ T cell signatures may capture immune processes beyond standard clinical and paraclinical markers. Variability in symptom onset, timing of disease presentation, and factors not captured by imaging could introduce confounders, potentially explaining why conventional parameters did not align with our findings.

Our results highlight the potential of CSF immune profiling to identify pathogenic T cell subsets with greater relevance for disease activity and progression. While T2 lesion load and oligoclonal bands aid in diagnosis, their prognostic value is limited—oligoclonal bands primarily distinguish MS from non-MS, and lesion burden correlates only moderately with long-term disability. Identifying CSF-resident immune signatures may improve patient stratification and treatment selection, particularly as many MS patients require therapy adjustments early in the disease course due to treatment failures.

To further validate these findings, experimental models could help clarify the role of chronic phase CD4+ T cells in sustaining neuroinflammation. Selective depletion assays in EAE and in vitro models may reveal mechanisms underlying T cell persistence, tissue retention and resistance to apoptosis. Combining CSF immune profiling with longitudinal clinical follow-up and experimental studies could yield more precise biomarkers for disease course and treatment response, ultimately advancing personalized therapy in MS.

Despite these promising results, our study has some limitations. The single-timepoint analysis of the transcriptomic and phenotypic states may not fully capture the dynamic nature of CD4+ T cells, particularly transcriptomically silent memory T cells. CSF cells also present handling challenges due to their poor viability ex vivo, which may lead to rapid cell decline. Ongoing research is exploring more robust protocols for handling CSF cells.^39^ Additionally, the methodological transfer from RNA expression patterns into epitope staining for flow cytometry requires sufficient protein expression and the availability of specific antibodies, which limits the inclusion of some cluster-defining markers. Consequently, some markers could not be incorporated into our flow cytometry panel due to the absence of appropriate antibodies or intracellular localization. A deeper integration of cell surface protein expression and transcription in CSF T cells of pwMS—linking surface marker expression with transcriptional programmes—could refine our understanding of immune dynamics in different disease phases. Future studies using CITE-Seq, which combines protein and RNA profiling at the single-cell level, may provide a more comprehensive characterization of MS1 and MS2 subtypes. Expanding spectral flow cytometry cohorts and applying unbiased bioinformatics approaches will be essential to uncover potential correlations between disease progression, immune signatures and treatment responses. Chronic phase markers are consistently and significantly enriched in MS-derived Tregs and CD4⁺ cells—especially within the CSF—supporting their role in disease persistence and progression.

Currently, the prediction on disease course is speculative due to small patient numbers in our cohort. It also remains to be elucidated, how the clinical relapse (as in most of our cases) or a remission influences the CSF cellularity. While our panel focuses primarily on CD4+ T cells, other CSF immune cells, such as B cells, warrant further analysis to gain a more comprehensive understanding of CSF cellularity in MS.

## Conclusion

This study demonstrates that in-depth characterization of CD4+ T cells via spectral flow cytometry reveals distinct inflammatory disease states in CSF of early MS patients. These findings underscore the importance of characterizing CSF cellularity across larger patient cohorts to uncover potential correlations between cellular markers and clinical markers, offering valuable insights for therapeutic implications. Establishing robust CSF handling and expanding marker panels to include additional immune cell types, such as B cells, will be instrumental in advancing our understanding of the cellular landscape in MS and other neuroinflammatory diseases.

## Supplementary Material

fcaf231_Supplementary_Data

## Data Availability

Any code used for data analysis in this study has been made available in the supplementary materials or through a publicly accessible repository. The RNA sequencing data generated from the mouse experimental autoimmune encephalomyelitis (EAE) model have been deposited in an open-access repository https://www.ncbi.nlm.nih.gov/geo/query/acc.cgi?acc=GSE156196 (Upload number: GSE156196). Due to ethical and informed consent restrictions, human flow cytometry data are not publicly available but can be shared upon reasonable request to the corresponding authors. Further methodological details, including data preprocessing and analysis scripts, can also be provided upon request.
