# Supplementary material for: Mapping CD4+ T cell diversity in CSF to identify endophenotypes of multiple sclerosis
Source: Brain Commun. 2025 Jun 10;7(3):fcaf231. doi: 10.1093/braincomms/fcaf231 (PMC12199765; doi:10.1093/braincomms/fcaf231)
Supplement: fcaf231_Supplementary_Data [file fcaf231_supplementary_data.zip › Supplementary_Figures.docx]

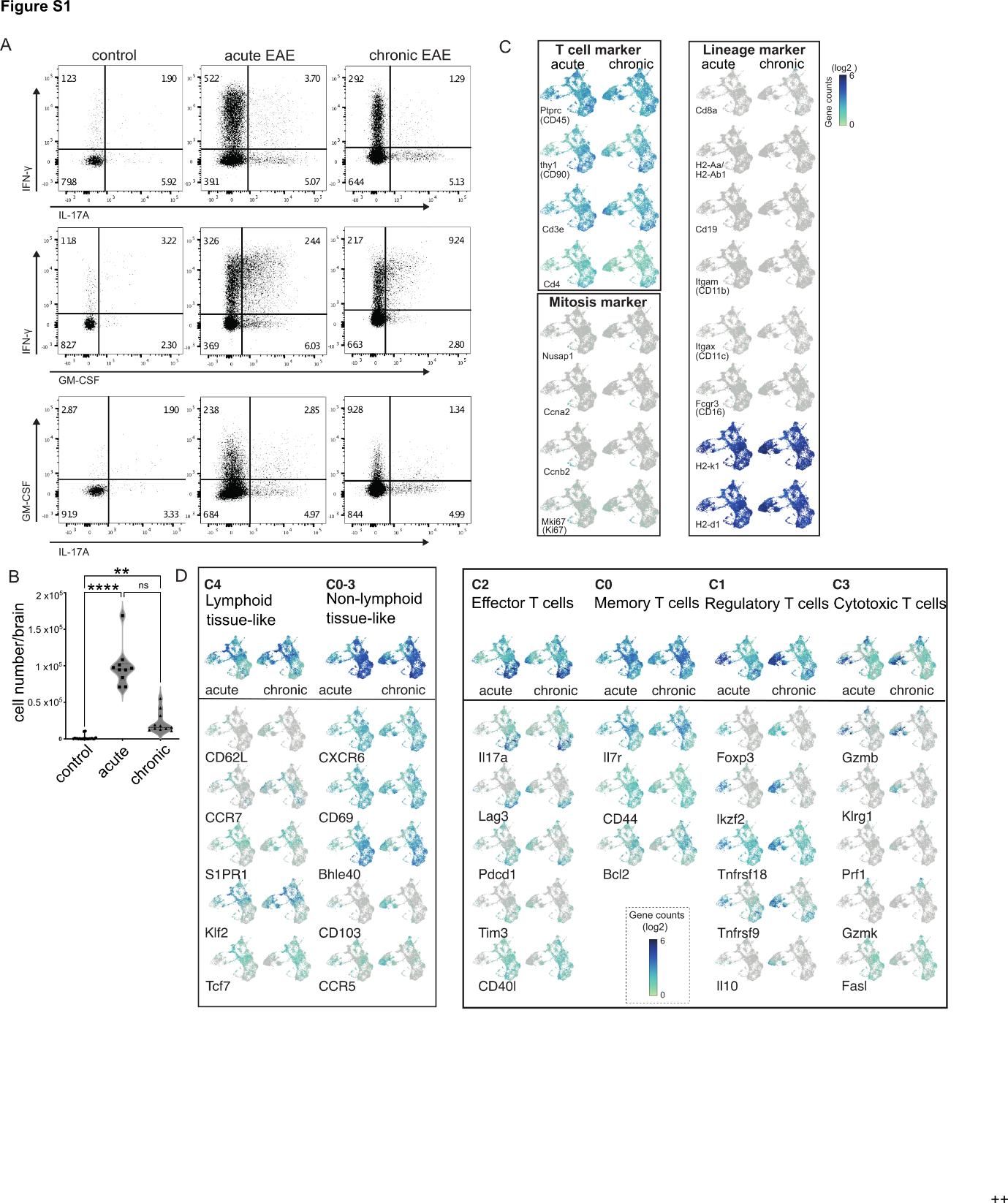


**Fig. S1 Characterization of CNS-isolated CD4+ T cells.**

(A) Cytokine profile of in vitro CD3/CD28-stimulated CNS CD45hi CD11b− CD4+ T cells in naïve, acute, and chronic phases of EAE. Compared to the naïve control group, a high proportion of CNS-isolated viable CD45hi CD11b− CD4+ T cells stained positive for IFN‐γ, IL-17A, and GM-CSF under EAE conditions. Data are concatenated from samples of at least 3 animals per condition (maximum of 12,000 dots shown). Experiments were repeated independently at least twice. Sample sizes: n=4 control; n=4 acute EAE; n=4 chronic EAE.

(B) Quantification of isolated CD4+ cells from the CNS (cells per mouse); statistical analysis performed using the Kruskal-Wallis test; ns=non-significant, *P < 0.05, **P < 0.01, ***P < 0.001, ****P < 0.0001**.

(C) Single-gene expression of individual CNS CD4+ T cells (represented as dots) visualized via UMAPs, with color-coded intensity indicating log2-transformed gene expression levels. Acute (left, n=2 (each 2 pooled mice) and chronic (right, n=2, (each 4 pooled mice) EAE samples are shown. Gene expression corresponding to conventional T cell differentiation phenotypes is visualized column-wise. Canonical markers identifying Th1, Th2, and Th17 phenotypes are presented, along with markers for CD4+ T cells, B cells, myeloid cells, innate immune cells, and mitosis.

(D) Column-wise summation of gene expression for acute (left) and chronic (right) EAE samples visualized via UMAPs with color-coded intensity (log2-transformed). Each dot represents a single CNS CD4+ T cell. Canonical markers are used to identify lymphoid tissue-like T cells, non-lymphoid tissue T cells, effector, memory, regulatory, and cytotoxic T cells. Single-gene expression is displayed via UMAPs with intensity color-coded (log2 [reads]). First row summation of all markers; rows 2-6: single markers. Sample sizes: acute EAE (left, n=2, pooled); chronic EAE (right, n=2, pooled). The colour scale indicates the intensity of gene expression (range: 0–6 average fold).

**
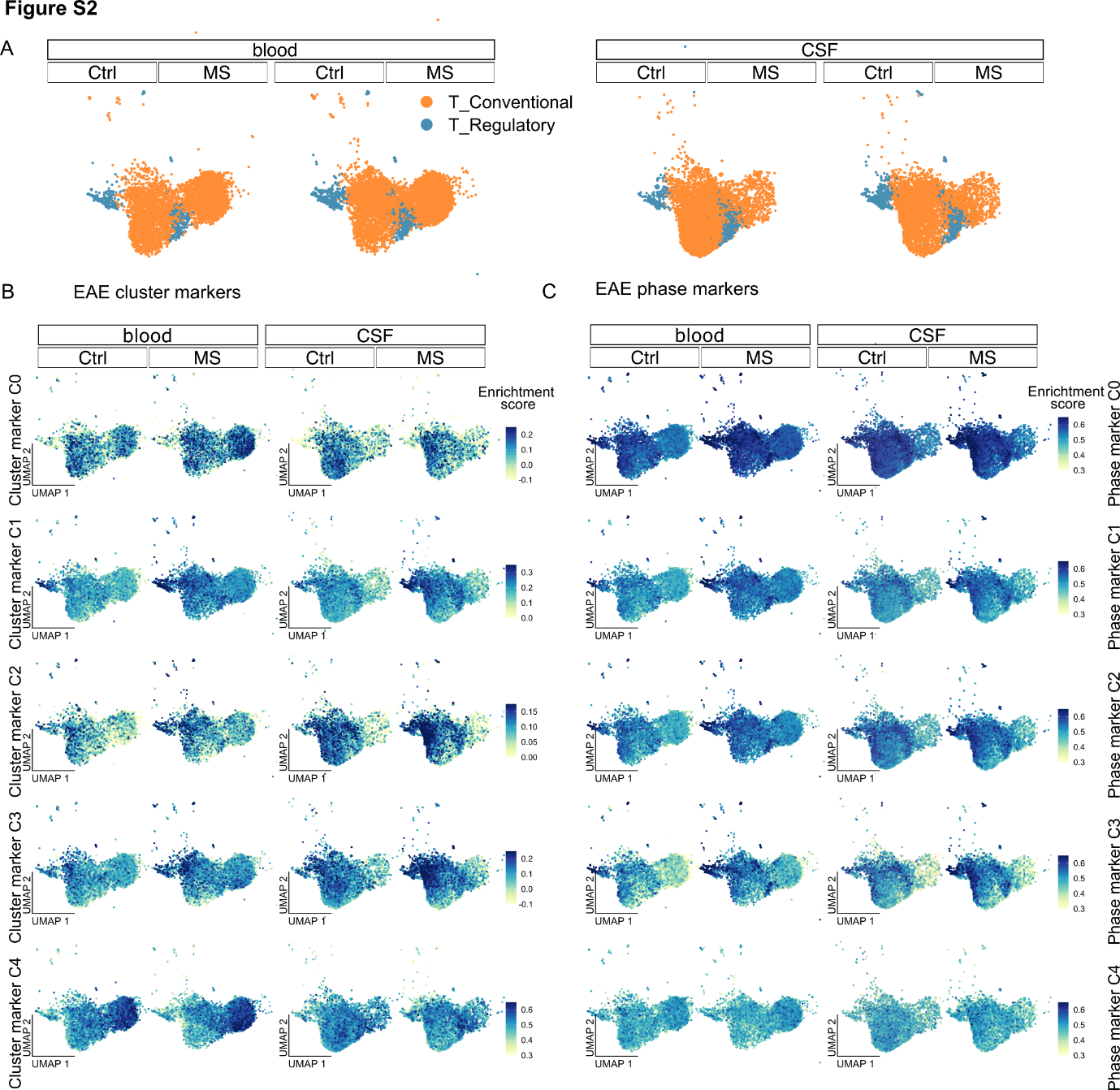
**

**Fig. S2 UMAP density plots showing cluster- and phase-specific gene expression in human CD4+ T cells (corresponding to the box-and-whisker plots in Figure 3)**

(A) UMAPs depicting conventional and regulatory CD4+ T cells in the dataset from Schafflick et al., which includes samples from both control individuals and people with MS, derived from CSF and blood.

(B) UMAPs with cluster-specific markers (C0 to C4) identified in the EAE experiment mapped onto the human datasets showing enrichment of EAE cluster-specific markers in human CD4+ T cells (control vs. MS and blood vs. CSF). C1 markers (Treg-associated) are most prominently enriched in MS Tregs, particularly in both blood and CSF, indicating a disease-specific regulatory T cell adaptation. C2 markers (effector-like) show a trend of enrichment in both Tcon and Tregs in the CSF compared to blood, but without strong disease-specific differences; C3 Markers(cytotoxic-like) are more enriched in MS vs. control, particularly in the CSF, suggesting a potential role in chronic neuroinflammation. C4 markers (lymphoid tissue-like) remain largely unchanged across conditions and compartments. Statistical analyses were performed using the Mann–Whitney U test, with Bonferroni correction applied for multiple comparisons (see Table S5 for full details).

(C) UMAPs showing enrichment of EAE (chronic vs. acute)-phase-specific markers in human CD4+ T cells. C0 to C3 phase markers show a strong enrichment in MS vs. control in both CSF and blood, with the most pronounced enrichment in Tregs, supporting their relevance in chronic neuroinflammation and immune persistence. C3 markers (cytotoxicity-associated) appear particularly increased in MS, both in blood and CSF. C4 phase markers do not show notable disease- or compartment-specific differences. Statistical analyses were performed using the Mann–Whitney U test, with Bonferroni correction applied for multiple comparisons (see Table S6 for full details).

**
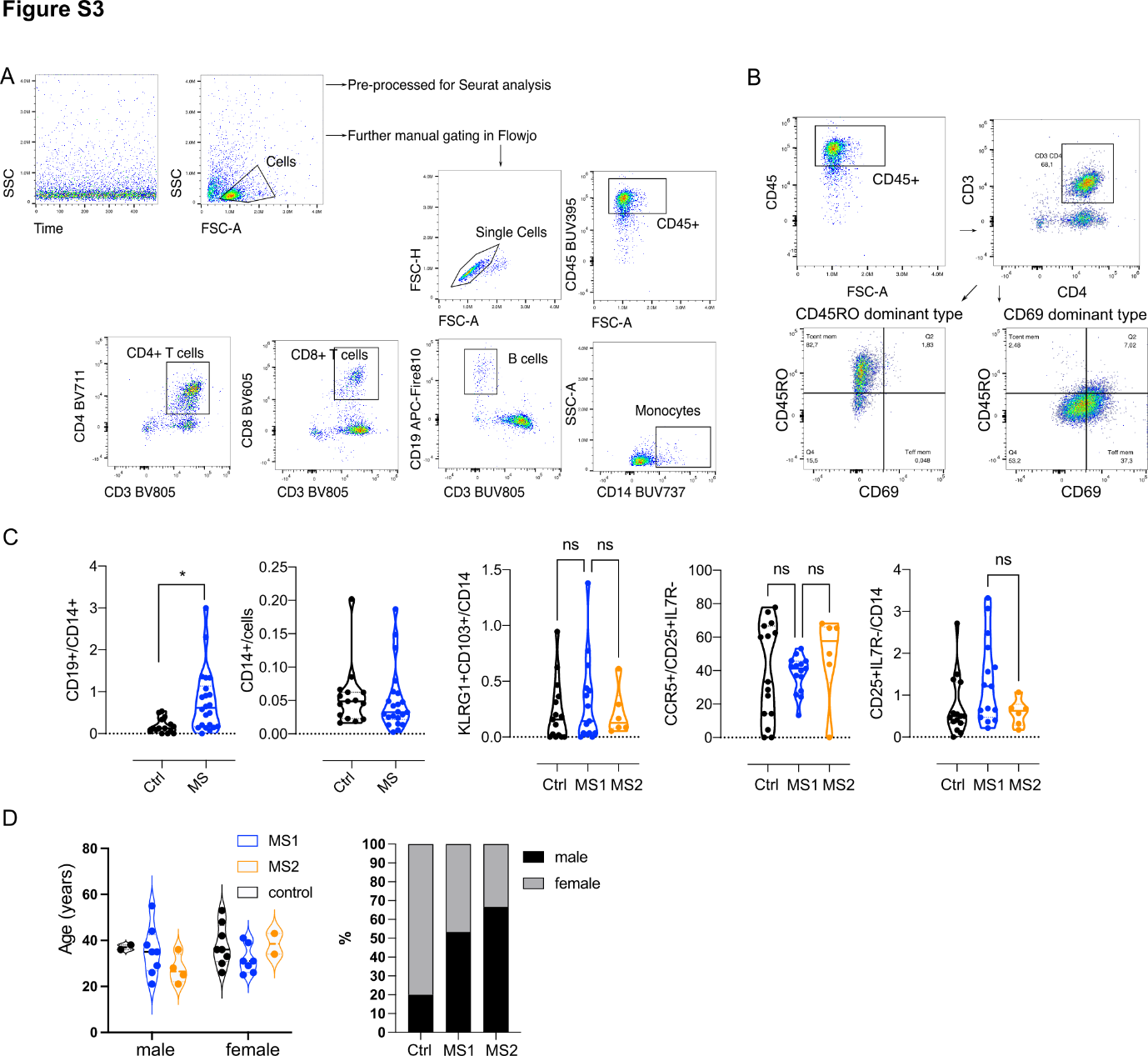
**

**Fig. S3 Gating strategy, manual analysis and patient characteristics.**

(A) Manual gating validation of CD4⁺ T cell subpopulations in CSF using FlowJo®, performed in both the discovery (MS = 6, controls = 5) and extended (total MS = 21, controls = 15) cohorts.

(B) The gating strategy confirms differences in T cell subset composition observed in clustering analyses, primarily driven by variation in CD45RO and CD69 expression. Despite variability in CSF cell counts and potential batch effects, patterns were consistent across samples, operators, and acquisition days.

(C) Quantitative analysis of CD19+/CD14+ ratios and CD14+ counts obtained through manual gating. Statistical analysis of key markers. Each data point represents one patient sample in groups as indicated. Data were tested for normal distribution using the Shapiro-Wilk test and analysed with one-way ANOVA or the Mann-Whitney test where appropriate. Significance levels are indicated as *P < 0.05.

(D) Scatter plots showing the absence of significant correlations between patient age or sex and CD4⁺ T cell subset distributions in CSF. These analyses support that observed immune signatures are independent of age or sex. Each data point represents one patient sample in groups as indicated.
