# Supplementary material for: Mapping CD4+ T cell diversity in CSF to identify endophenotypes of multiple sclerosis
Source: Brain Commun. 2025 Jun 10;7(3):fcaf231. doi: 10.1093/braincomms/fcaf231 (PMC12199765; doi:10.1093/braincomms/fcaf231)
Supplement: fcaf231_Supplementary_Data [file fcaf231_supplementary_data.zip › Supplementary_Table_S2.docx]

# Supplementary Table S2

| Cluster | gene | significance | reference |  |
| --- | --- | --- | --- | --- |
| 0 | Ramp3 | adrenomedullin receptor involved in calcitonin signaling | Liverani et al., 2012 | ^1^ |
| 0 | Actn2 | Actinin alpha 2 which is a cytostructural protein recently described to be co-regulated with Serpinb1a in tissue resident T cells | Tan et al, 2019 | ^2^ |
| 0 | Neurl3 | E3 ubiquitin-protein ligase, an endosomal protein with as yet unknown function in T cells |  |  |
| 1 | Ikzf4 | EOS has been shown to be essential for Treg suppressor function | Gokhale et al, 2019 | ^3^ |
| 1 | Itgae/  CD103 | integrin tissue resident marker, usually associated with CD8+ TRM | Mackay et al., 2013 | ^4^ |
| 1 | Ncmap | non-compact myelin associated protein the function of which has not yet been described in Tregs |  |  |
| 1 | Areg | Amphiregulin, epidermal growth factor family, recently described as a potential anti-neurotoxic astrogliosis factor produced by brain-derived Treg after experimental stroke | Ito et al, 2019 | ^5^ |
| 1 | Neb | nebulin which is an actin-binding protein described in muscle cells but not immune cells |  |  |
| 1 | Cd74 | MHC class II associated chaperone protein which has a crucial role in MHC-II peptide loading and protein trafficking | Su et al, 2017 | ^6^ |
| 1 | Tnfrsf13b | transmembrane activator and cyclophilin ligand interactor (TACI), a receptor for the B cell chemokines BAFF and APRIL which have been described as promoting Treg survival by anti-apoptotic downstream signaling | Tai et al, 2019 | ^7^ |
| 1 | Fabp5 | fatty acid binding protein which has been recently described as expressed on Tregs in a tissue inflammation context | Layland et al, 2010 | ^8^ |
| 1 | Tnfrsf1b | TNFR2 described as relevant for Treg function in EAE | Atretkhany et al, 2018 | ^9^ |
| 1 | Fgl2 | secreted fibrinogen-like protein found to be essential for Treg function in an inflammatory bowel disease model and the presence of which in the brain is associated with tumor progression in murine glioma | Bartczak et al, 2017;  Yan et al, 2019 | ^10,11^ |
| 2 | Klrc1 | Natural Killer Group protein 2A (NKG2A) which is it involved in atypical MHC-I (HLA-E) recognition (negative regulation) primarily in NK Cells |  |  |
| 2 | Nmrk1 | metabolic enzyme essential for NAD+ synthesis and energy metabolism |  |  |
| 2 | Il1r1 | marker of encephalitogenic Th17 cells in EAE | Komuczki et al, 2019 | ^12^ |
| 2 | Bhlhe40 | EAE relevant transcription factor and marker of tissue resident effector cells | Lin et al, 2016;  Li et al, 2019 | ^13,14^ |
| 2 | Cxcr6 | tissue resident T cells’ chemokine receptor | Wein et al., 2019 | ^15^ |
| 2 | Nfkb1 | encoding inflammation-related transcription factors |  |  |
| 2 | Rel |  |  |  |
| 2 | Furin | pro-protein convertase which modulates TCR-activated transactivation and NF-κB signaling | Ortutay et al, 2015 | ^16^ |
| 3 | Dusp2 | direct STAT3 phosphatase involved in the downregulation of Th17 cell differentiation | Lu et al, 2015 | ^17^ |
| 3 | Itgb1 | encoding for integrin beta 1, associates with high cytotoxicity in CD8+ T cells | Nicolet et al., 2020,  Bauer et al., 2009 | ^18,19^ |
| 4 | Lef1 | typical lymphoid-tissue marker upregulated in chronic EAE | Steinke et al, 2014 | ^20^ |
| 4 | Cd9 | tetraspanin family relevant for immune cell interactions | Jones et al, 2011 | ^21^ |
| 4 | Gimap6 | anti-apoptotic function in T cells | Ho & Tsai, 2017 | ^22^ |

References:

1. Liverani E, McLeod JD, Paul C. Adrenomedullin receptors on human T cells are glucocorticoid-sensitive. *Int Immunopharmacol*. 2012;14(1):75-81. doi:10.1016/j.intimp.2012.06.011

2. Tan L, Sandrock I, Odak I, et al. Single-Cell Transcriptomics Identifies the Adaptation of Scart1+ Vγ6+ T Cells to Skin Residency as Activated Effector Cells. *Cell Rep*. 2019;27(12):3657-3671.e4. doi:10.1016/j.celrep.2019.05.064

3. Gokhale AS, Gangaplara A, Lopez-Occasio M, Thornton AM, Shevach EM. Selective deletion of Eos (Ikzf4) in T-regulatory cells leads to loss of suppressive function and development of systemic autoimmunity. *J Autoimmun*. Published online July 8, 2019:102300. doi:10.1016/j.jaut.2019.06.011

4. Mackay LK, Rahimpour A, Ma JZ, et al. The developmental pathway for CD103(+)CD8+ tissue-resident memory T cells of skin. *Nat Immunol*. 2013;14(12):1294-1301. doi:10.1038/ni.2744

5. Ito M, Komai K, Mise-Omata S, et al. Brain regulatory T cells suppress astrogliosis and potentiate neurological recovery. *Nature*. 2019;565(7738):246-250. doi:10.1038/s41586-018-0824-5

6. Su H, Na N, Zhang X, Zhao Y. The biological function and significance of CD74 in immune diseases. *Inflamm Res Off J Eur Histamine Res Soc Al*. 2017;66(3):209-216. doi:10.1007/s00011-016-0995-1

7. Tai YT, Lin L, Xing L, et al. APRIL signaling via TACI mediates immunosuppression by T regulatory cells in multiple myeloma: therapeutic implications. *Leukemia*. 2019;33(2):426-438. doi:10.1038/s41375-018-0242-6

8. Layland LE, Mages J, Loddenkemper C, et al. Pronounced phenotype in activated regulatory T cells during a chronic helminth infection. *J Immunol Baltim Md 1950*. 2010;184(2):713-724. doi:10.4049/jimmunol.0901435

9. Atretkhany KSN, Mufazalov IA, Dunst J, et al. Intrinsic TNFR2 signaling in T regulatory cells provides protection in CNS autoimmunity. *Proc Natl Acad Sci U S A*. 2018;115(51):13051-13056. doi:10.1073/pnas.1807499115

10. Bartczak A, Zhang J, Adeyi O, et al. Overexpression of fibrinogen-like protein 2 protects against T cell-induced colitis. *World J Gastroenterol*. 2017;23(15):2673-2684. doi:10.3748/wjg.v23.i15.2673

11. Yan J, Zhao Q, Gabrusiewicz K, et al. FGL2 promotes tumor progression in the CNS by suppressing CD103+ dendritic cell differentiation. *Nat Commun*. 2019;10(1):448. doi:10.1038/s41467-018-08271-x

12. Komuczki J, Tuzlak S, Friebel E, et al. Fate-Mapping of GM-CSF Expression Identifies a Discrete Subset of Inflammation-Driving T Helper Cells Regulated by Cytokines IL-23 and IL-1β. *Immunity*. 2019;50(5):1289-1304.e6. doi:10.1016/j.immuni.2019.04.006

13. Lin CC, Bradstreet TR, Schwarzkopf EA, et al. IL-1-induced Bhlhe40 identifies pathogenic T helper cells in a model of autoimmune neuroinflammation. *J Exp Med*. 2016;213(2):251-271. doi:10.1084/jem.20150568

14. Li C, Zhu B, Son YM, et al. The Transcription Factor Bhlhe40 Programs Mitochondrial Regulation of Resident CD8+ T Cell Fitness and Functionality. *Immunity*. 2019;51(3):491-507.e7. doi:10.1016/j.immuni.2019.08.013

15. Wein AN, McMaster SR, Takamura S, et al. CXCR6 regulates localization of tissue-resident memory CD8 T cells to the airways. *J Exp Med*. 2019;216(12):2748-2762. doi:10.1084/jem.20181308

16. Ortutay Z, Oksanen A, Aittomäki S, Ortutay C, Pesu M. Proprotein convertase FURIN regulates T cell receptor-induced transactivation. *J Leukoc Biol*. 2015;98(1):73-83. doi:10.1189/jlb.2A0514-257RR

17. Lu D, Liu L, Ji X, et al. The phosphatase DUSP2 controls the activity of the transcription activator STAT3 and regulates TH17 differentiation. *Nat Immunol*. 2015;16(12):1263-1273. doi:10.1038/ni.3278

18. Bauer M, Brakebusch C, Coisne C, et al. Beta1 integrins differentially control extravasation of inflammatory cell subsets into the CNS during autoimmunity. *Proc Natl Acad Sci U S A*. 2009;106(6):1920-1925. doi:10.1073/pnas.0808909106

19. Nicolet BP, Guislain A, van Alphen FPJ, et al. CD29 identifies IFN-γ-producing human CD8+ T cells with an increased cytotoxic potential. *Proc Natl Acad Sci U S A*. 2020;117(12):6686-6696. doi:10.1073/pnas.1913940117

20. Steinke FC, Yu S, Zhou X, et al. TCF-1 and LEF-1 act upstream of Th-POK to promote the CD4(+) T cell fate and interact with Runx3 to silence Cd4 in CD8(+) T cells. *Nat Immunol*. 2014;15(7):646-656. doi:10.1038/ni.2897

21. Jones EL, Demaria MC, Wright MD. Tetraspanins in cellular immunity. *Biochem Soc Trans*. 2011;39(2):506-511. doi:10.1042/BST0390506

22. Ho CH, Tsai SF. Functional and biochemical characterization of a T cell-associated anti-apoptotic protein, GIMAP6. *J Biol Chem*. 2017;292(22):9305-9319. doi:10.1074/jbc.M116.768689
