# Supplementary material for: Mapping CD4+ T cell diversity in CSF to identify endophenotypes of multiple sclerosis
Source: Brain Commun. 2025 Jun 10;7(3):fcaf231. doi: 10.1093/braincomms/fcaf231 (PMC12199765; doi:10.1093/braincomms/fcaf231)
Supplement: fcaf231_Supplementary_Data [file fcaf231_supplementary_data.zip › Supplementary_Table_S3.docx]

# Supplementary Table S3

| Cluster | Gene | Role | Reference | |
| --- | --- | --- | --- | --- |
| 0 | Ifitm2,  Ifitm3 | interferon-(gamma-)induced transmembrane proteins primarily described in innate immune responses against viruses.  promotes survival of CD8+ T cells at the site of viral inflammation and has been described as sensitizing resting CD4+ T cells for Th1 reaction on activation | Bedford et al., 2019; Yánez et al., 2019 | ^1,2^ |
| 1 | Ikzf2 | Transcription factor in Treg | Fu et al., 2012 | ^3^ |
| 1 | Il1rl1 | ST2, IL-33 receptor able to boost Foxp3 dependent programs | Peine et al., 2016 | ^4^ |
| 1 | Cd81 | tetraspanin involved in immune synapse formation, controlling T cell activation | Jones et al., 2011 | ^5^ |
| 1 | Cd83 | suppressive Treg cell function in different autoimmune models incl. EAE | Reinwald et al., 2008 | ^6^ |
| 1 | Bmyc | brain expressed myelocytomatosis oncogene, identified in an scRNA-seq of nTreg | Zemmour et al., 2018 | ^7^ |
| 1 | Izumo1r | folate receptor with role in Lef1/Tcf1-dependent immunosuppression of Treg cells | Xing et al., 2019 | ^8^ |
| 1 | Serpina3 | plasma serine protease inhibitor (alpha-3-antitrypsin) reported to be expressed in cytotoxic T cells and (similarly to Gzmb) predominantly expressed in clusters 1 and 3 | van Aalderen et al., 2017 | ^9^ |
| 1 | Klrg1 | marker of effector CD8+ T cells in non-lymphoid tissues and activated/effector Treg cells in non-lymphoid organs e.g. the gut | Yang et al., 2019 | ^10^ |
| 1 | Psen2 | amyloid cleaving enzyme most studied in the context of familial Alzheimer’s disease | Sannerud et al., 2016 | ^11^ |
| 1 | Pglyrp1 | peptidoglycan recognition protein described in innate immune responses of polymorphonuclear leukocytes and also on tumor-infiltrating Tregs | Downs-Canner et al., 2017 | ^12^ |
| 1 | Glrx | glutaredoxin which is a glutathione-dislulfide oxidoreductase with antioxidant functions and expressed in activated Treg | Sugimoto et al., 2006 | ^13^ |
| 1 | Tspan32 | tumor suppressor gene with antiproliferative function in T cells through restriction of IL-2 expression | Tarrant et al., 2002 | ^14^ |
| 1 | Matk | megakaryocyte-associated tyrosine kinase protein which might also control T cell proliferation | Chow et al., 1994 | ^15^ |
| 1 | Lrrc32 | leucine-rich repeat-containing protein 32 (also known as Garp) which is a transmembrane receptor for TGF-beta1 thereby controlling activation of Treg | Tran et al., 2009 | ^16^ |
| 1 | CD27 | A TNF receptor family member, immune checkpoint molecule i through interaction with CD70 on B cells | Hendriks et al., 2000 | ^17^ |
| 1 | Tnfrsf9 | Known to be involved in the fine tuning of regulatory T cell responses and development are also enriched on cluster 1 cells and may play a role in T-cell survival and differentiation within the inflamed CNS | Vasanthakumar et al., 2017 | ^18^ |
| 1 | Tnfrsf4 |  |  |  |
| 1 | Tnfrsf18 |  |  |  |
| 1 | Ighm | Encoding for the constant region of heavy chain of IgM which plays an essential role as a B cell receptor, in Notch cleavage and in regulatory immune responses | Tournoy et al., 2004 | ^19^ |
| 1 | Igav | integrin alpha chain V which, essential factor for Th1 cell migration in inflamed skin | Overstreet et al., 2013 | ^20^ |
| 1 | Arl5a | Golgi-associated GTP binding protein, endosome-to-Golgi transport | Rosa-Ferreira et al., 2015 | ^21^ |
| 1 | Snx18 | sorting nexin 18, involved in clathrin-mediated endocytosis | Park et al., 2010 | ^22^ |
| 2 | Tff1 | delaying cell cycle progression | Bossenmeyer-Pourié et al., 2002 | ^23^ |
| 2 | Igfbp7 | Inhibitor of cell proliferation | Heesch et al., 2010 | ^24^ |
| 2 | Il1r2 | scavenging pro-inflammatory cytokine signaling of Il1 | Shimizu et al., 2015 | ^25^ |
| 2 | Rgs16 | negative regulator of G-protein mediated chemokine signaling | Lippert et al., 2003 | ^26^ |
| 2 | Gem | GTP-Binding Mitogen-Induced T-Cell Protein | Maguire et al., 1994 | ^27^ |
| 2 | Stx11 | Syntaxin 11, required for protein trafficking from endo(lyso)some to cell membrane. Stx11 dysfunction leads to familial hemophagocytic lymphohistiocytosis, a severe hyperinflammation syndrome | Halimani et al., 2014 | ^28^ |
| 2 | Ptms | Involved in RNA transcription | Vareli et al., 2000 | ^29^ |
| 2 | Litaf | (Lipopolysaccharide-induced tumor necrosis factor-alpha factor) inducer of TNFa gene expression | Myokai et al., 1999 | ^30^ |
| 2 | Tmem176a | Is a Th17/Rorc dependent cation channel, which is important for Th17 activation | Drujont et  al., 2016 | ^31^ |
| 2 | Tnfsf11 | Encoding Rankl, essential for pathogenicity of T cells in EAE | Guerrini et  al., 2015 | ^32^ |
| 2 | Rbpja | A Notch signaling transcription factor important for T cell survival | Tanigaki et  al., 2004 | ^33^ |
| 2 | Phlda1/  TDAG51 | Implicated in programmed cell death in T cells | Oberg et al.,  2004 | ^34^ |
| 2 | Rgcc | A complement-sensitive regulator of cell cycle progression | Saigusa et  al., 2007 | ^35^ |
| 2 | Zeb2 | (CD40lg-like expression ) a transcription factor that cooperates with Tbet in CD8+ T cells for terminal differentiation into effector cells | Gautam et  al., 2019 | ^36^ |
| 2 | Ramp1 | A gene induced downstream of CGRP relevant for Th17 activation and EAE and Nr4a1—critical for Th17/Th1 modulation in EAE | Mikami et  al., 2012 | ^37^ |
| 3 | Gzmk | Potential effector molecule of CNS-pathogenic T cells | Herich et al.,  2019 | ^38^ |
| 3 | Nkg7 | Encoding for the natural killer cell-associated transmembrane protein natural killer cell granule protein 7 (NKG7). NKG7 has been poorly characterized but seems to play a role for lytic granule function | Medley et al.,  1996 | ^39^ |
| 3 | Ccl5 | Chemokine identified on brain TRM | Steinbach et  al., 2019 | ^40^ |
| 3 | Ccr5 | Chemokine receptor relevant for EAE | Gu et al.,  2016 | ^41^ |
| 3 | Ly6c2 | transmembrane protein which has not yet been characterized in T cells |  |  |
| 3 | Ifit1 | Are RNA-binding, antiviral proteins | Fensterl and Sen, 2011 | ^42^ |
| 3 | Ifit3 | Are RNA-binding, antiviral proteins | Fensterl and Sen, 2011 | ^42^ |
| 3 | Isg15 | Is an ubiquitin-like antiviral protein with a role in the inhibition of virus entry and replication | Villarroya-Beltri et al., 2017 | ^43^ |
| 3 | Zbp1 | antiviral protein that binds to foreign DNA and functions as a pathogen sensor | Kuriakose and Kanneganti, 2018 | ^44^ |
| 3 | Zfp36l2 | gene involved in cell cycle progression | Galloway et al., 2016 | ^45^ |
| 3 | Ms4a4b | member of the CD20 family that also has a role in controlling cell cycle progression | Xu et al., 2010 | ^46^ |
| 4 | Slamf6 | transmembrane protein reported to increase TCR signaling | Dragovich et al., 2019 | ^47^ |
| 4 | Tcf7 | transcriptional activator of thymocyte differentiation | Mielke et al.,  2019 | ^48^ |
| 4 | Socs3 | negative regulator of IL-6 family cytokine signaling | Linossi et al., 2018 | ^49^ |
| 4 | Dusp10 | inactivator of MAP kinases and constrictor of pro-inflammatory activities | Yamamoto et  al., 2018 | ^50^ |
| 4 | Trib2 | pseudokinase and negative regulator of thymocytes | Liang et al.,  2016 | ^51^ |
| 4 | Prkca | protein kinase C alpha and reportedly relevant for TCR down-regulation | von Essen et al., 2006 | ^52^ |
| 4 | Klf3 | transcriptional repressor involved in manifold biological processes | Pearson et al., 2011 | ^53^ |
| 4 | Hmgn1 | high mobility group nucleosome binding protein 1 an alarmin relevant for activation of dendritic cells | Yang et al., 2012 | ^54^ |
| 4 | Reflnb | Refilin-B, a perinuclear structure protein involved in cytoskeleton formation with reported co-expression with S1pr1 | Tabula Muris Consortium et al., 2018 | ^55^ |
| 4 | Adk | adenosine kinase, generation of extracellular adenosine, unknown roles in inflammatory T cells |  |  |
| 4 | Ramp3 | E receptor activity modifying protein 3 involved in adrenomedullin receptor signaling through shuttling and modulation of calcitonin receptor-like receptors | Liverani et al., 2012 | ^56^ |

References:

1. Bedford JG, O’Keeffe M, Reading PC, Wakim LM. Rapid interferon independent expression of IFITM3 following T cell activation protects cells from influenza virus infection. *PLOS ONE*. 2019;14(1):e0210132. doi:10.1371/journal.pone.0210132

2. Yánez DC, Sahni H, Ross S, et al. IFITM proteins drive type 2 T helper cell differentiation and exacerbate allergic airway inflammation. *Eur J Immunol*. 2019;49(1):66-78. doi:10.1002/eji.201847692

3. Fu W, Ergun A, Lu T, et al. A multiply redundant genetic switch “locks in” the transcriptional signature of regulatory T cells. *Nat Immunol*. 2012;13(10):972-980. doi:10.1038/ni.2420

4. Peine M, Marek RM, Löhning M. IL-33 in T Cell Differentiation, Function, and Immune Homeostasis. *Trends Immunol*. 2016;37(5):321-333. doi:10.1016/j.it.2016.03.007

5. Jones EL, Demaria MC, Wright MD. Tetraspanins in cellular immunity. *Biochem Soc Trans*. 2011;39(2):506-511. doi:10.1042/BST0390506

6. Reinwald S, Wiethe C, Westendorf AM, et al. CD83 expression in CD4+ T cells modulates inflammation and autoimmunity. *J Immunol Baltim Md 1950*. 2008;180(9):5890-5897. doi:10.4049/jimmunol.180.9.5890

7. Zemmour D, Zilionis R, Kiner E, Klein AM, Mathis D, Benoist C. Single-cell gene expression reveals a landscape of regulatory T cell phenotypes shaped by the TCR. *Nat Immunol*. 2018;19(3):291-301. doi:10.1038/s41590-018-0051-0

8. Xing S, Gai K, Li X, et al. Tcf1 and Lef1 are required for the immunosuppressive function of regulatory T cells. *J Exp Med*. 2019;216(4):847-866. doi:10.1084/jem.20182010

9. van Aalderen MC, van den Biggelaar M, Remmerswaal EBM, et al. Label-free Analysis of CD8+ T Cell Subset Proteomes Supports a Progressive Differentiation Model of Human-Virus-Specific T Cells. *Cell Rep*. 2017;19(5):1068-1079. doi:10.1016/j.celrep.2017.04.014

10. Yang J, Ramadan A, Reichenbach DK, et al. Rorc restrains the potency of ST2+ regulatory T cells in ameliorating intestinal graft-versus-host disease. *JCI Insight*. 2019;4(5). doi:10.1172/jci.insight.122014

11. Sannerud R, Esselens C, Ejsmont P, et al. Restricted Location of PSEN2/γ-Secretase Determines Substrate Specificity and Generates an Intracellular Aβ Pool. *Cell*. 2016;166(1):193-208. doi:10.1016/j.cell.2016.05.020

12. Downs-Canner S, Berkey S, Delgoffe GM, et al. Suppressive IL-17A+Foxp3+ and ex-Th17 IL-17AnegFoxp3+ Treg cells are a source of tumour-associated Treg cells. *Nat Commun*. 2017;8:14649. doi:10.1038/ncomms14649

13. Sugimoto N, Oida T, Hirota K, et al. Foxp3-dependent and -independent molecules specific for CD25+CD4+ natural regulatory T cells revealed by DNA microarray analysis. *Int Immunol*. 2006;18(8):1197-1209. doi:10.1093/intimm/dxl060

14. Tarrant JM, Groom J, Metcalf D, et al. The absence of Tssc6, a member of the tetraspanin superfamily, does not affect lymphoid development but enhances in vitro T-cell proliferative responses. *Mol Cell Biol*. 2002;22(14):5006-5018. doi:10.1128/mcb.22.14.5006-5018.2002

15. Chow LM, Jarvis C, Hu Q, et al. Ntk: a Csk-related protein-tyrosine kinase expressed in brain and T lymphocytes. *Proc Natl Acad Sci*. 1994;91(11):4975-4979. doi:10.1073/pnas.91.11.4975

16. Tran DQ, Andersson J, Wang R, Ramsey H, Unutmaz D, Shevach EM. GARP (LRRC32) is essential for the surface expression of latent TGF-beta on platelets and activated FOXP3+ regulatory T cells. *Proc Natl Acad Sci U S A*. 2009;106(32):13445-13450. doi:10.1073/pnas.0901944106

17. Hendriks J, Gravestein LA, Tesselaar K, van Lier RA, Schumacher TN, Borst J. CD27 is required for generation and long-term maintenance of T cell immunity. *Nat Immunol*. 2000;1(5):433-440. doi:10.1038/80877

18. Vasanthakumar A, Liao Y, Teh P, et al. The TNF Receptor Superfamily-NF-κB Axis Is Critical to Maintain Effector Regulatory T Cells in Lymphoid and Non-lymphoid Tissues. *Cell Rep*. 2017;20(12):2906-2920. doi:10.1016/j.celrep.2017.08.068

19. Tournoy J, Bossuyt X, Snellinx A, et al. Partial loss of presenilins causes seborrheic keratosis and autoimmune disease in mice. *Hum Mol Genet*. 2004;13(13):1321-1331. doi:10.1093/hmg/ddh151

20. Overstreet MG, Gaylo A, Angermann BR, et al. Inflammation-induced interstitial migration of effector CD4^+^ T cells is dependent on integrin αV. *Nat Immunol*. 2013;14(9):949-958. doi:10.1038/ni.2682

21. Rosa-Ferreira C, Christis C, Torres IL, Munro S. The small G protein Arl5 contributes to endosome-to-Golgi traffic by aiding the recruitment of the GARP complex to the Golgi. *Biol Open*. 2015;4(4):474-481. doi:10.1242/bio.201410975

22. Park J, Kim Y, Lee S, et al. SNX18 shares a redundant role with SNX9 and modulates endocytic trafficking at the plasma membrane. *J Cell Sci*. 2010;123(Pt 10):1742-1750. doi:10.1242/jcs.064170

23. Bossenmeyer-Pourié C, Kannan R, Ribieras S, et al. The trefoil factor 1 participates in gastrointestinal cell differentiation by delaying G1-S phase transition and reducing apoptosis. *J Cell Biol*. 2002;157(5):761-770. doi:10.1083/jcb200108056

24. Heesch S, Schlee C, Neumann M, et al. BAALC-associated gene expression profiles define IGFBP7 as a novel molecular marker in acute leukemia. *Leukemia*. 2010;24(8):1429-1436. doi:10.1038/leu.2010.130

25. Shimizu K, Nakajima A, Sudo K, et al. IL-1 receptor type 2 suppresses collagen-induced arthritis by inhibiting IL-1 signal on macrophages. *J Immunol Baltim Md 1950*. 2015;194(7):3156-3168. doi:10.4049/jimmunol.1402155

26. Lippert E, Yowe DL, Gonzalo JA, et al. Role of regulator of G protein signaling 16 in inflammation-induced T lymphocyte migration and activation. *J Immunol Baltim Md 1950*. 2003;171(3):1542-1555. doi:10.4049/jimmunol.171.3.1542

27. Maguire J, Santoro T, Jensen P, Siebenlist U, Yewdell J, Kelly K. Gem: an induced, immediate early protein belonging to the Ras family. *Science*. 1994;265(5169):241-244. doi:10.1126/science.7912851

28. Halimani M, Pattu V, Marshall MR, et al. Syntaxin11 serves as a t-SNARE for the fusion of lytic granules in human cytotoxic T lymphocytes. *Eur J Immunol*. 2014;44(2):573-584. doi:10.1002/eji.201344011

29. Vareli K, Frangou-Lazaridis M, van der Kraan I, Tsolas O, van Driel R. Nuclear Distribution of Prothymosin α and Parathymosin: Evidence That Prothymosin α Is Associated with RNA Synthesis Processing and Parathymosin with Early DNA Replication. *Exp Cell Res*. 2000;257(1):152-161. doi:10.1006/excr.2000.4857

30. Myokai F, Takashiba S, Lebo R, Amar S. A novel lipopolysaccharide-induced transcription factor regulating tumor necrosis factor α gene expression: Molecular cloning, sequencing, characterization, and chromosomal assignment. *Proc Natl Acad Sci U S A*. 1999;96(8):4518. doi:10.1073/pnas.96.8.4518

31. Drujont L, Lemoine A, Moreau A, et al. RORγt + cells selectively express redundant cation channels linked to the Golgi apparatus. *Sci Rep*. 2016;6(1):1-13. doi:10.1038/srep23682

32. Guerrini MM, Okamoto K, Komatsu N, et al. Inhibition of the TNF Family Cytokine RANKL Prevents Autoimmune Inflammation in the Central Nervous System. *Immunity*. 2015;43(6):1174-1185. doi:10.1016/j.immuni.2015.10.017

33. Tanigaki K, Tsuji M, Yamamoto N, et al. Regulation of alphabeta/gammadelta T cell lineage commitment and peripheral T cell responses by Notch/RBP-J signaling. *Immunity*. 2004;20(5):611-622. doi:10.1016/s1074-7613(04)00109-8

34. Oberg HH, Sipos B, Kalthoff H, Janssen O, Kabelitz D. Regulation of T-cell death-associated gene 51 (TDAG51) expression in human T-cells. *Cell Death Differ*. 2004;11(6):674-684. doi:10.1038/sj.cdd.4401407

35. Saigusa K, Imoto I, Tanikawa C, et al. RGC32, a novel p53-inducible gene, is located on centrosomes during mitosis and results in G2/M arrest. *Oncogene*. 2007;26(8):1110-1121. doi:10.1038/sj.onc.1210148

36. Gautam S, Fioravanti J, Zhu W, et al. The transcription factor c-Myb regulates CD8+ T cell stemness and antitumor immunity. *Nat Immunol*. 2019;20(3):337. doi:10.1038/s41590-018-0311-z

37. Mikami N, Watanabe K, Hashimoto N, et al. Calcitonin gene-related peptide enhances experimental autoimmune encephalomyelitis by promoting Th17-cell functions. *Int Immunol*. 2012;24(11):681-691. doi:10.1093/intimm/dxs075

38. Herich S, Schneider-Hohendorf T, Rohlmann A, et al. Human CCR5high effector memory cells perform CNS parenchymal immune surveillance via GZMK-mediated transendothelial diapedesis. *Brain J Neurol*. 2019;142(11):3411-3427. doi:10.1093/brain/awz301

39. Medley QG, Kedersha N, O’Brien S, et al. Characterization of GMP-17, a granule membrane protein that moves to the plasma membrane of natural killer cells following target cell recognition. *Proc Natl Acad Sci U S A*. 1996;93(2):685-689. doi:10.1073/pnas.93.2.685

40. Steinbach K, Vincenti I, Kreutzfeldt M, et al. Brain-resident memory T cells represent an autonomous cytotoxic barrier to viral infection. *J Exp Med*. 2016;213(8):1571-1587. doi:10.1084/jem.20151916

41. Gu SM, Park MH, Yun HM, et al. CCR5 knockout suppresses experimental autoimmune encephalomyelitis in C57BL/6 mice. *Oncotarget*. 2016;7(13):15382. doi:10.18632/oncotarget.8097

42. Fensterl V, Sen GC. The ISG56/IFIT1 Gene Family. *J Interferon Cytokine Res*. 2011;31(1):71-78. doi:10.1089/jir.2010.0101

43. Villarroya-Beltri C, Guerra S, Sánchez-Madrid F. ISGylation - a key to lock the cell gates for preventing the spread of threats. *J Cell Sci*. 2017;130(18):2961-2969. doi:10.1242/jcs.205468

44. Kuriakose T, Kanneganti TD. ZBP1: Innate Sensor Regulating Cell Death and Inflammation. *Trends Immunol*. 2018;39(2):123-134. doi:10.1016/j.it.2017.11.002

45. Galloway A, Saveliev A, Łukasiak S, et al. RNA-binding proteins ZFP36L1 and ZFP36L2 promote cell quiescence. *Science*. 2016;352(6284):453-459. doi:10.1126/science.aad5978

46. Xu H, Yan Y, Williams MS, et al. MS4a4B, a CD20 Homologue in T Cells, Inhibits T Cell Propagation by Modulation of Cell Cycle. *PLOS ONE*. 2010;5(11):e13780. doi:10.1371/journal.pone.0013780

47. Dragovich MA, Adam K, Strazza M, Tocheva AS, Peled M, Mor A. SLAMF6 clustering is required to augment T cell activation. *PLoS ONE*. 2019;14(6). doi:10.1371/journal.pone.0218109

48. Mielke LA, Liao Y, Clemens EB, et al. TCF-1 limits the formation of Tc17 cells via repression of the MAF-RORγt axis. *J Exp Med*. 2019;216(7):1682-1699. doi:10.1084/jem.20181778

49. Linossi EM, Calleja DJ, Nicholson SE. Understanding SOCS protein specificity. *Growth Factors Chur Switz*. 2018;36(3-4):104-117. doi:10.1080/08977194.2018.1518324

50. Yamamoto T, Endo Y, Onodera A, et al. DUSP10 constrains innate IL-33-mediated cytokine production in ST2hi memory-type pathogenic Th2 cells. *Nat Commun*. 2018;9(1):4231. doi:10.1038/s41467-018-06468-8

51. Liang KL, O’Connor C, Veiga JP, McCarthy TV, Keeshan K. TRIB2 regulates normal and stress-induced thymocyte proliferation. *Cell Discov*. 2016;2:15050. doi:10.1038/celldisc.2015.50

52. von Essen M, Nielsen MW, Bonefeld CM, et al. Protein kinase C (PKC) alpha and PKC theta are the major PKC isotypes involved in TCR down-regulation. *J Immunol Baltim Md 1950*. 2006;176(12):7502-7510. doi:10.4049/jimmunol.176.12.7502

53. Pearson RCM, Funnell APW, Crossley M. The mammalian zinc finger transcription factor Krüppel-like factor 3 (KLF3/BKLF). *IUBMB Life*. 2011;63(2):86-93. doi:10.1002/iub.422

54. Yang D, Postnikov YV, Li Y, et al. High-mobility group nucleosome-binding protein 1 acts as an alarmin and is critical for lipopolysaccharide-induced immune responses. *J Exp Med*. 2012;209(1):157-171. doi:10.1084/jem.20101354

55. Tabula Muris Consortium, Overall coordination, Logistical coordination, et al. Single-cell transcriptomics of 20 mouse organs creates a Tabula Muris. *Nature*. 2018;562(7727):367-372. doi:10.1038/s41586-018-0590-4

56. Liverani E, McLeod JD, Paul C. Adrenomedullin receptors on human T cells are glucocorticoid-sensitive. *Int Immunopharmacol*. 2012;14(1):75-81. doi:10.1016/j.intimp.2012.06.011
