# Supplementary material for: Mapping CD4+ T cell diversity in CSF to identify endophenotypes of multiple sclerosis
Source: Brain Commun. 2025 Jun 10;7(3):fcaf231. doi: 10.1093/braincomms/fcaf231 (PMC12199765; doi:10.1093/braincomms/fcaf231)
Supplement: fcaf231_Supplementary_Data [file fcaf231_supplementary_data.zip › Supplementary_Table_S7.docx]

**Supplement Table S7**

| **Group** | **Age** | **Sex** | **Diagnosis** |
| --- | --- | --- | --- |
| "MS1" | 35 | m | MS |
|  | 21 | m | MS |
|  | 31 | f | MS |
|  | 31 | f | ON/CIS |
|  | 35 | m | CIS |
|  | 38 | m | ON/CIS |
|  | 29 | f | MS |
|  | 26 | m | MS |
|  | 55 | m | MS |
|  | 25 | f | MS |
|  | 44 | m | MS |
|  | 26 | f | MS |
|  | 41 | f | MS |
|  | 29 | m | MS |
|  | 39 | f | ON/CIS |
| "MS2" | 36 | m | RIS |
|  | 21 | m | MS |
|  | 28 | m | MS |
|  | 43 | f | MS |
|  | 34 | f | CIS |
|  | 25 | m | MS |
| „Control“ | 26 | f | IIH |
|  | 33 | f | IIH |
|  | 36 | m | Non-inflammatory |
|  | 30 | f | IIH |
|  | 48 | f | IIH |
|  | 36 | f | IIH |
|  | 42 | f | Non-inflammatory |
|  | 38 | m | Non-inflammatory |
|  | 36 | f | Non-inflammatory |
|  | 53 | f | IIH |
